# The impact of regular sauerkraut consumption on the human gut microbiota: a crossover intervention trial

**DOI:** 10.1186/s40168-024-02016-3

**Published:** 2025-02-12

**Authors:** Nelly Schropp, Alexander Bauer, Virginie Stanislas, Kun D. Huang, Till-Robin Lesker, Agata Anna Bielecka, Till Strowig, Karin B. Michels

**Affiliations:** 1 Institute for Prevention and Cancer Epidemiology, Faculty of Medicine and Medical Center, University of Freiburg, Freiburg, 79110 Germany; 2 Department of Microbial Immune Regulation, Helmholtz Centre for Infection Research, Brunswick, 38124 Germany; 3 Center for Individualized Infection Medicine (CiiM), a joint venture between the Hannover Medical School (MHH), Helmholtz Centre for Infection Research (HZI), Hannover, 30625 Germany

**Keywords:** Sauerkraut, Fermented food, Gut microbiome, Pasteurization, Short-chain fatty acids, SCFA, Diversity, Shotgun metagenomics

## Abstract

**Background:**

Sauerkraut is a fermented food that has been suspected to have a beneficial impact on the gut microbiome, but scientific evidence is sparse. In this crossover intervention trial with 87 participants (DRKS00027007), we investigated the impact of daily consumption of fresh or pasteurized sauerkraut for 4 weeks on gut microbial composition and the metabolome in a healthy study population.

**Results:**

Using shotgun metagenomic sequencing, we observed changes in single bacterial species following fresh and pasteurized sauerkraut consumption. More pronounced changes were observed in the pasteurized sauerkraut intervention arm. Only pasteurized sauerkraut consumption increased serum short-chain fatty acids (SCFAs).

**Conclusions:**

The gut microbiome of healthy individuals is rather resilient to short-term dietary interventions even though single species might be affected by sauerkraut consumption.

Video Abstract

**Supplementary Information:**

The online version contains supplementary material available at 10.1186/s40168-024-02016-3.

## Introduction

Microbial genes add an estimated 100 times more genes to our human gene catalog, conferring additional physiological properties not encoded in the human DNA [1]. Of note, human gut bacterial diversity has decreased dramatically with the adoption of modern lifestyles [2], and a “shrinking” or “disappearing” gut microbiota has been correlated with an increased risk of numerous chronic diseases including obesity [2–6].

Diet provides a direct route to influence the intestinal microbiota [7]. Due to their long history of consumption in many cultures, fermented foods are promising candidates for reintroducing important microbial interactions that humans have co-evolved with over thousands of years, which may be effective in preventing modern diseases [8–10]. Fermented foods are significant sources of viable lactic acid-producing bacteria (LAB), many known to promote gastrointestinal health as potential “probiotics” [11, 12]. Probiotics are “live microorganisms that, when administered in adequate amounts, confer a health benefit on the host” [13]. The regular consumption of fermented foods has been related to phylogenetic differences in gut microbial composition and might impact the microbial metabolome in healthy individuals [14]. However, due to a great variety in study designs and outcomes, the diversity of fermented food-specific microbes, and the highly individual composition and dynamic nature of the intestinal microbiota, little is known about the impact of fermented foods on the gut microbiome [15–19]. Indeed, it is plausible that there are significant inter-individual variations in food-microbiome interactions [20].

Most studies on the health-promoting capabilities of fermented foods have hitherto focused on fermented dairy products [14, 17]. Salt-based fermented vegetables like sauerkraut indeed contain a significantly greater diversity of microbes than milk-based foods [16], with sauerkraut being the product of the microbial fermentation of cabbage dominated by LAB [12, 21]. Sauerkraut has been suggested to improve symptoms of irritable bowel syndrome (IBS) patients and significantly impact their gut microbiota in a study including 34 participants [22]. However, there is only limited evidence for the modulatory and health-promoting capacities of sauerkraut and it is unknown whether healthy individuals respond differently compared to IBS patients [15, 23].

Gut microbiota-derived products important in IBS pathophysiology are SCFAs, which may be effective in treating IBS symptoms, but this has not been sufficiently evaluated [24]. SCFAs are important bacterial metabolites produced mainly from dietary fiber that mediate dietary microbial impacts on the host [25]. They are physiologically important metabolites also produced by the microbiota in the colon, the most densely populated human body site, where they are mostly rapidly absorbed by the host [26–28]. Acetic acid, propionic acid, and butyric acid, derived from carbohydrate fermentation, are the most abundant SCFAs in the gut, where they exist in their anionic form [26, 29]. Interestingly, in the study of IBS patients cited above, pasteurized sauerkraut appeared to have similar effects to fresh sauerkraut. Pasteurization has replaced fermentation as the main practice for food preservation and has been extensively applied since the nineteenth century to stop the growth of microbes. This process can destroy or inactivate microorganisms, although it does not necessarily eliminate their physiological properties or products like SCFAs [30–32].

In this study, we used shotgun metagenomic sequencing and ultra-high-performance liquid chromatography/tandem accurate mass spectrometry (UHPLC/MS/MS) to analyze the influence of the daily consumption of 100 g fresh and pasteurized sauerkraut for 4 weeks, on the composition, function, and metabolic output of the fecal bacterial microbiota in healthy adults. We aimed to address the question of whether fresh and pasteurized sauerkraut shifts the microbial composition and SCFA levels of healthy individuals. We also explored whether responders and non-responders were identifiable in our population, because BMI, sex, dietary fiber intake, and age have been associated with differences in microbial profiles [33–35].

## Materials and methods

### Study design and procedure

This study is based on a monocentric trial with a randomized crossover design conducted at the Institute for Prevention and Cancer Epidemiology, Faculty of Medicine and Medical Center of the University of Freiburg, Germany. The University of Freiburg's ethical review committee approved the project, which was registered at the German Clinical Trials Register (DRKS; https://www.drks.de identifier: DRKS00027007). All participants provided written informed consent before any data or samples were collected.

**Fig. 1:**
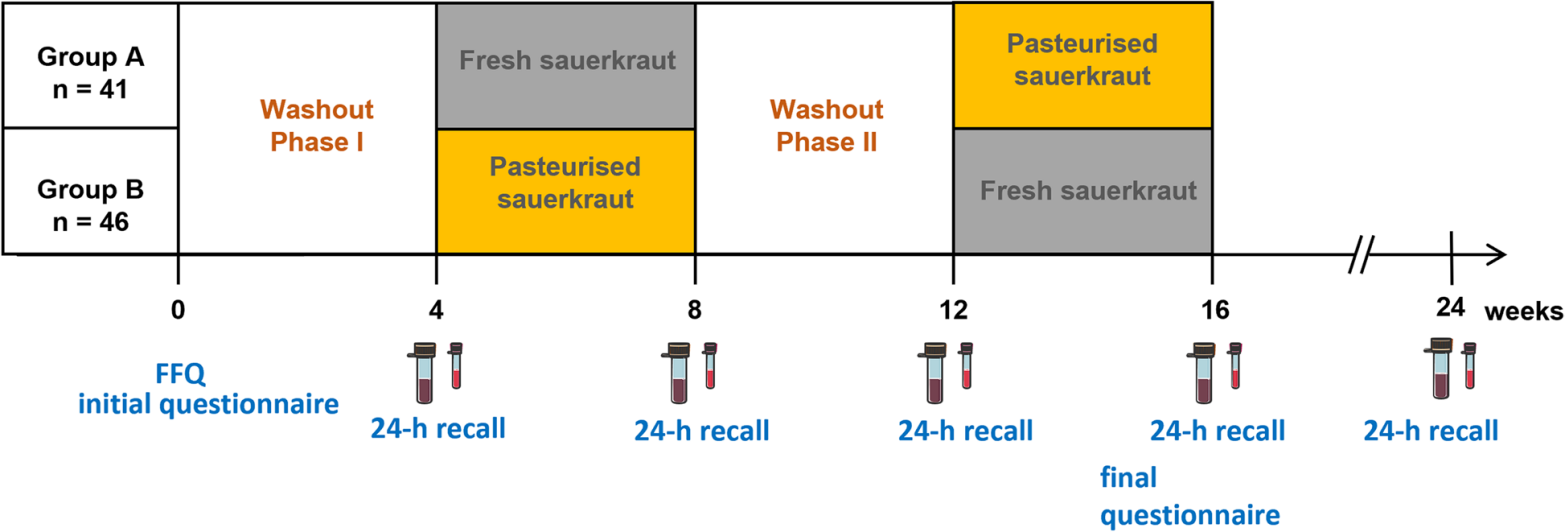
Overview of the study procedure; vials indicate stool and serum sampling, 24 h recalls of consumed foods the day before stool sampling were recorded online; FFQ = food frequency questionnaire

The study included two intervention phases, each of 4 weeks duration (see Fig. 1), during which each participant consumed 100 g of fresh or pasteurized sauerkraut daily. Before each intervention phase, participants underwent a 4-week washout phase. Washout periods served to eliminate the effects of previously consumed probiotics and fermented foods or pre- and probiotic supplements, including carry-over effects from the first to the second intervention. There are still no clear standards for how long a gut microbiome-targeted intervention period should last, but there is evidence that dietary effects on the gut microbiota can be observed within days [36]. A time period of 4 weeks has been shown to be sufficient to wash out short-term intervention effects of prebiotics following a significant increase in fecal SCFA levels and probiotic yogurt in other trials [37, 38]. Participants were instructed to avoid the consumption of other fermented foods and food with added lactic acid bacteria during both washout and intervention phases (16 weeks overall). Particular emphasis was placed on fermented dairy products like yogurt, kefir, or crème fraiche, other fermented vegetables, and fermented beverages like kombucha. Participants were allowed to eat cheese as we intended to limit dietary restrictions to enhance compliance and reduce interferences with the participants’ regular lifestyle and nutritional habits. Alcohol consumption was restricted to one standard drink per day. During the study, participants were instructed to omit lifestyle changes like starting sports programs or changing their diet. They were provided a log to record deviations from our guidelines or incidences like diseases or medications during the study. Adherence to the dietary guidelines was additionally ensured based on food-frequency questionnaires (FFQs) and 24-h recalls at the end of all study phases. The Human Study Site of the German Institute for Nutritional Research Potsdam-Rehbrücke, Potsdam, Germany, provided electronic dietary questionnaires. We invited all participants to a follow-up appointment 8 weeks after the second intervention to assess long-term effects. Biospecimens were collected at the end of each phase, including the 8 weeks follow-up visits, yielding five assessments per participant.

### Participants

Healthy male and female study participants were recruited between October 2021 and August 2022 in the region of Freiburg. Invitations to participate in the study were posted on the University Hospital intranet and published in local newspapers, Facebook, small advertising online portals, gyms, and grocery stores. Flyers were mainly handed out to pharmacies and general practitioner surgeries, but also to individuals willing to distribute them in their personal or professional environment. Exclusion criteria were smoking, diabetes mellitus, hypertension, acute or chronic gastrointestinal disease, cancer, cardiovascular incidences, or antibiotic treatments more than 3 months before the beginning of the study, a history of severe gastrointestinal surgery, and considerable dietary restrictions including veganism or eating disorders (vegetarians were included). Upon their first visit, participants were screened regarding our inclusion criteria through a questionnaire. Body height and weight were taken. Participants fulfilling the criteria were randomly allocated to group A or B, starting their first intervention either with fresh or pasteurized sauerkraut, respectively.

Forty-one men and 65 women were recruited in the study. Two men and 16 women dropped out throughout the trial due to time constraints, illness, and/or antibiotic treatment. Three participants completed only one of the two interventions but were included in our analysis. The reasons for their dropouts were illness in two cases and a move to another city in one case. This left 49 women, 48 of whom completed all aspects of the study, and 38 men, 36 of whom fully completed the trial. One participant took sulfamethoxazol, trimethoprim, and cefurax (antibiotics) during the follow-up phase, so we excluded his final specimen from the analysis.

### Intervention

We selected a local manufacturer (“EDEN”, Hügli Nahrungsmittel GmbH, Radolfzell, Germany) producing organic fresh and pasteurized pure sauerkraut all year round. We thereby intended to improve the comparability of the products by circumventing the potential influences of additives such as starch, sugar, or wine, pesticides, and discrepancies in cabbage cultivation, transport, or processing conditions. The fresh sauerkraut was supplied in a fermentation-active glass and contained a starter culture. The manufacturer’s nutritional information is presented in Table 1. The mean pH of three spot-checked sauerkraut samples, determined with pH indicator strips, was 3.4 ± 0.06 for the fresh and 3.8 ± 0.06 for the pasteurized version. The pasteurized sauerkraut was the result of spontaneous fermentation and additionally contained juniper berries, which was acceptable because they are usually not consumed and can be removed from the sauerkraut easily. According to the manufacturer, who had no knowledge that we used their sauerkraut for a scientific study, pasteurization involved a 5-min. heat treatment at 75 °C. The required amount of sauerkraut was purchased and delivered to our institute and stored at 4 °C before handing it out to the participants on their first day of each intervention phase. Participants were instructed to keep the provided sauerkraut at home in the refrigerator and weigh each 100 g portion per day by themselves. They were allowed to heat the pasteurized, but not the fresh sauerkraut.

**Table 1:** Nutritional information provided by the manufacturer for both sauerkraut variants used. These values are subject to natural variation

	Fresh	Pasteurized
Energy	107 kj/25 kcal	124 kj/kcal
Fat	0 g	0 g
Carbohydrates thereof sugars	3.5 g 3.0 g	4.0 g 1.2 g
Fiber	2.5 g	3.3 g
Proteins	1 g	0.9 g
Salt	0.8 g	1.2 g
Vitamin C	15 mg	12 mg

### Specimen collection and assessments

For stool self-collection, participants were provided with a standardized fecal collection kit, including detailed instructions on how to use it. At each time point of collection, a stool collector was used to obtain two stool specimens: one native specimen for SCFA analysis and one stabilized with 96% ethanol for DNA sequencing. Participants were instructed to keep the specimens at home in the refrigerator before transporting them to our institute in a cooling bag. The morning after the last day of each study phase, participants presented themselves at our institute, donating their self-collected stool specimens and a 12-h fasting serum sample. Blood serum was allowed to clot in the S-monovette (Sarstedt AG & Co, Nümbrecht, Germany) for 30 min at room temperature before centrifugation at 2000 × *g* for 10 min. The pH of native stool was assessed using indicator strips pH-Fix 4.5–10.0 (Carl Roth GmbH + Co. KG, Karlsruhe, Germany). Determination of fecal pH was not attainable in cases of excessively dry stool, as observed in 21 instances. Aliquots of stool and serum specimens were stored at – 80 °C before being sent to specialized laboratories for further analyses.

The fecal collection kit also contained a questionnaire addressing the texture of the stool using the Bristol Stool Form Chart and the mean defecation frequency during the previous week. The Bristol Stool Form Chart is a widely used scale that divides stool consistency into seven types, ranging from severe constipation (type 1) to severe diarrhea (type 7) [39]. We determined body weight at each appointment to calculate the body mass index (BMI). If participants reported disease-related gastrointestinal symptoms like diarrhea, nausea, or constipation up to 3 days before stool collection in their log, the respective stool type was not considered in the Bristol Stool Form analysis (resulting in 6 single time point exclusions).

## Laboratory analyses

### Metagenomic sequencing

Shotgun metagenomic analysis was performed at the Helmholtz Center for Infection Research, Braunschweig, Germany. A stool specimen stabilized in 96% ethanol as well as three aliquots of sauerkraut juice from three different batches of fresh and pasteurized sauerkraut, respectively, were shipped on dry ice at the end of the study to identify contained bacterial species and map abundant genes.

Aliquots were conserved in QuickSepar tubes. DNA was extracted using the ZymoBIOMICS 96 MagBead DNA Kit (D4308). Libraries were constructed using Illumina DNA PCR-Free Prep Kit without size selection. Libraries were sequenced on the Illumina NovaSeq 6000 S4 system with a target depth of 25,000,000 reads per sample.

All metagenomic samples in this study were first subjected to raw sequence preprocessing using the BBMap quality control pipeline (sourceforge.net/projects/bbmap/), which removed read bases with quality score below 10 and reads from human host (the Ensembl masked human genome GRCh38) and phiX contamination (*minid* = *0.95 maxindel* = *3 bwr* = *0.16 bw* = *12 quickmatch fast minhits* = *2 qtrim* = *rl trimq* = *10*). Afterward, for each sample, the species-level microbial community was profiled, and the abundance of each microbial member was quantified using MetaPhlAn 4 with -t rel_ab_w_read_stats –force –s*b* [40]. For functional profiling, bwa-mem was used with the default setting to align the preprocessed reads against the integrated gene catalog (IGC), a comprehensive database comprising 9,879,896 genes specific to the human gut microbiome functionality [41, 42]. The aligned reads were normalized in copies per million (CPM), a generic analog of the transcripts per million (TPM) unit of RNA-Seq. Subsequently, KEGG annotations [43] were assigned to aligned IGC genes using KofamKOALA [44]. Lastly, MinPath was used for summarizing and integrating KEGG-annotated genes into pathways [45].

The 193 KEGG orthologues (KOs) related to SCFA production assessed in this study were selected based on the work of Zhang and colleagues [46].

### SCFA analysis

Metabolon Inc., Morrisville, USA, performed ultra-high-performance liquid chromatography/tandem accurate mass spectrometry (UHPLC/MS/MS) to assess SCFA levels in serum and native stool specimens.

Human samples were analyzed for eight short-chain fatty acids: acetic acid (C2), propionic acid (C3), iso-butyric acid (C4), butyric acid (C4), 2-methyl-butyric acid (C5), iso-valerate (C5), valerate (C5) and caproate (hexanoate, C6) by LC–MS/MS (Metabolon Method TAM135: “LC–MS/MS Method for the Quantitation of Short Chain Fatty Acid (C2 to C6) in Human Feces” and Metabolon Method TAM148: “LC–MS/MS Method for the Quantitation of Short Chain Fatty Acid (C2 to C6) in Human Plasma and Serum”).

Human samples were spiked with stable labeled internal standards, homogenized, and subjected to protein precipitation with an organic solvent. After centrifugation, an aliquot of the supernatant was derivatized. The reaction mixture was diluted, and an aliquot was injected into an Agilent 1290/AB Sciex QTrap 5500 LC MS/MS system (feces) or 1290/SCIEX QTrap 5500 LC MS/MS system (serum) equipped with a C18 reversed-phase UHPLC column. The mass spectrometer was operated in negative mode using electrospray ionization (ESI). The peak area of the individual analyte product ions was measured against the peak area of the product ions of the corresponding internal standards. Quantitation was performed using a weighted linear least squares regression analysis generated from fortified calibration standards prepared concurrently with study samples. LC–MS/MS raw data was collected and processed using AB SCIEX software Analyst 1.7.3 and processed using SCIEX OS-MQ software v3.0 (feces) and SCIEX OS-MQ software v3.1.6 (serum).

### Statistical analyses

Intra-sample and inter-sample microbial variation in the fecal microbiomes were quantified using several α- and β-diversity measures, respectively, all calculated on relative abundances. Regarding α-diversity, we analyzed a broad set of indicators: diversity (Shannon and inversed Simpson), richness (observed and Hill), evenness (Pielou and Simpson), dominance (core abundance and DBP) and rarity (log modulo skewness). β-diversity was analyzed using unweighted UniFrac distances, Bray–Curtis dissimilarities, and a UMAP (Uniform Manifold Approximation and Projection) projection [47] as a visualization tool based on dimension reduction. Based on the pair-wise distances in the 2D UMAP graph being moderately to strongly correlated with the respective Bray–Curtis dissimilarities (Spearman correlation 0.63), we concluded that the UMAP visualization led to a meaningful graphical representation of the overall β-diversity. If not explicitly stated, all β-diversity measures led to similar findings as the displayed results (see Supplementary Fig. S6).

Inferential statistics was used to evaluate the effect of the fresh and pasteurized sauerkraut interventions on the following measures: (A) the values of the outlined 9 α-diversity measures, (B) the concentration of 8 SCFAs in blood serum (B.1) and in the stool samples (B.2), and the abundance of (C) 362 bacteria species [unit: relative abundance], (D) 2,785 SCFA-related genes [TPM], and (E) 100 pathways [TPM] (limited to pathways related to metabolism, genetic information processing or cellular processes). The latter three components were limited to sets with relevant abundances, i.e. species (or genes/pathways) with a minimum abundance of 0.01% (or 10 TPM) in at least 10% of the samples. Apart from the SCFAs, all measures were based on the stool samples. Genes and pathways should be interpreted with some caution since, on average, only 6.3% of processed reads were able to be assigned to IGC genes with known KEGG functional characterization and 1% of reads could be eventually integrated into KEGG pathways due to the limitation of the existing database. SCFAs were analyzed separately in stool and blood since these two SCFA concentration measurements are practically uncorrelated in our data (max. Spearman correlation of 0.13, see Supplementary Fig. S15).

For every measure, we individually estimated the intervention effects based on a (generalized) linear mixed regression model (GLMM) [48], utilizing all measurements apart from the follow-up, and with a random intercept controlling for the intra-subject correlation of each participant’s measurements. Intervention effects are estimated based on a categorical variable (with values “baseline”, “post-fresh intervention”, and “post-pasteurized intervention”) as the main independent variable. Control variables include participants’ sex (“male”, “female”), age (centered around 50 years), baseline BMI (centered around 25), and the study phase (“intervention phase 1”, “intervention phase 2”). The estimated control variable effects can be found in Supplementary Figs. S8 to S10. Carry-over effects between post-intervention measurements and the following baseline are not explicitly accounted for in the regression models since the study design implicitly builds on reasonably long washout phases that prevent any relevant carry-over effects. Applying the hypothesis test for the existence of a carry-over effect proposed by Wellek and Blettner [49] to our central parameters Shannon α-diversity (*p* = 0.8378), acetic acid (in blood, *p* = 0.5887), and butyric acid (in stool, *p* = 0.3188) did not lead to significant results.

To ensure the use of an adequate model structure for each measure, a model selection approach is applied. For measures in groups (A) and (B) we estimated each regression as (1) normal regression, (2) normal regression on log(y), and (3) gamma regression. Models in groups (C) to (E) were each estimated as (1) Normal regression, and (2) Normal regression on log(y + 1). For species/genes/pathways whose abundances in groups (C) to (E) were 0% in at least 10% of all samples, the two models were estimated as zero-inflated mixed models [50], to account for this presence of excess zero values. For each individual measure in groups (A) to (E), the best-fitting model was selected based on the share of explained deviance. Model assumptions were visually checked based on the residual distribution (for all models in (A) and (B) and for selected models in (C)–(E)). No model showed relevant deviation from the model assumptions.

Additionally to overall intervention effects, subgroup-specific intervention effects were estimated individually for the five subgroup definitions: baseline Shannon α-diversity (“ ≥ 3.85” vs. “ < 3.85”, based on the median as cutpoint), sex (“male” vs. “female”), age (“ ≥ 50 years” vs. “ < 50 years”),baseline BMI (“ ≥ 25” vs. 25″) and daily fiber intake (“ ≥ 30 g” vs. “ < 30 g”). For each of these definitions, and all models apart from the pathway models (E), each measure’s final model was re-estimated with subgroup-specific intervention effects.

Due to the high number of significance tests for the taxonomic analyses, we account for multiple tests by calculating *q*-values (i.e., corrected *p*-values) for all respective intervention effects. Benjamini–Hochberg correction [51] is applied separately in the taxonomic model groups (A), (C), (D), and (E), and in these again separately, once for all main intervention effects, and once for all subgroup-specific intervention effects. To facilitate interpretation, we will use the term “effect” in the following to talk about associations which are significant after correction for multiple testing (*q*-value threshold 0.1). Additionally, to discuss further tendencies found in the data with an exploratory character, we will use the term “trend” for non-significant associations (i.e., *q*-value ≥ 0.1), whose *p*-values *before correction* for multiple testing are below 0.05. For the separate SCFA analyses (B) we do not account for multiple testing and talk about “effects” referring to the classical significance threshold of 0.05.

We randomly selected 5% of biospecimen to be measured in duplicate to estimate the measuring inaccuracy. The intra-class correlation coefficient (ICC) for every species was estimated based on its relative abundance. ICCs were calculated based on linear mixed regression models with the sample indicator as a fixed effect and the binary indicator ‘original/duplicate measurement’ as a random intercept. Reliability was high, with an average ICC of 2% (2.5% and 97.5% quantiles over all species: 0% and 13%).

All analyses were performed using the open-source statistical software R [52]. α-diversity measures were calculated with package “mia” [53]. The UMAP representation was calculated using the function “calculateUMAP” from package “scater” [54], and is visualized in Fig. 3 along with subgroup-specific ‘data ellipses’ (summarizing the 2D distribution) [55] drawn with function “stat_ellipse” from package “ggplot2” [56]. Regression models were estimated with function “gam” from package “mgcv” [57], and zero-inflated models with function “lme.zig” from package “NBZIMM” [58].

## Results

Our study included 87 participants, 84 of which completed all aspects of the study, aged 21–69 with a BMI between 18.1 and 30.8 kg/m^2^. Table 2 presents the characteristics of our study population. The mean age was substantially higher in intervention group A. However, due to the crossover design of our study (in which every participant serves as his/her own control), this difference should not confound our results.

**Table 2:** Characteristics of our study population by intervention groups. Groups A and B received the fresh and pasteurized sauerkraut first, respectively. The distribution of sex is reported as “absolute frequency (relative frequency)”, and age and baseline BMI as “mean ± standard deviation (minimum–maximum)”

Characteristic	Full sample	Group a	Group b
Participants	87	41	46
Sex—male	38 (43.7%)	18 (43.9%)	20 (43.5%)
Sex—female	49 (56.3%)	23 (56.1%)	26 (56.5%)
Mean age ± std. dev. (min.–max.)	44.4 ± 14.0 (21–69)	47.5 ± 12.9 (23–69)	41.6 ± 14.4 (21–67)
Mean BMI ± std. dev. (min.–max.)	24.1 ± 3.0 (18.1–30.8)	24.1 ± 3.0 (18.1–29.7)	24.1 ± 3.0 (18.1–30.8)

### Bacterial species in the fresh and pasteurized sauerkraut

Shotgun metagenomic sequencing of the two sauerkraut types used in our study revealed that the fresh sauerkraut was dominated by *Lacticaseibacillus paracasei* (95.5% mean relative abundance, see Supplementary Fig. S1). As the sauerkraut was contained in a fermentation-active glass, the microbial composition changed over time. After 7 weeks of cooled storage—still before the expiration date—*Limosilactobacillus reuteri* was the dominant species (60.8% mean relative abundance). Other species identified at low abundance include *Leuconostoc mesenteroides, Weissella cibaria, Lactococcus lactis*, and *Bradyrhizobium species 17–4.* The pasteurized sauerkraut contained no living bacteria and little bacterial DNA. Thus, we obtained no reliable data on the present species (Supplementary Table S1).

### Tolerance and digestive impact of sauerkraut consumption

Consumption of 100 g fresh and pasteurized sauerkraut for 4 weeks was well received and tolerated by our study population (95% and 90% contentment after fresh and pasteurized intervention, respectively, assessed by a questionnaire). We did not observe a significant change according to the Bristol Stool Form, defecation frequency, or fecal pH after consuming either sauerkraut (Supplementary Fig. S2).

### The impact of sauerkraut on the fecal microbiome

#### α-diversity: within-sample differences before and after the interventions

**Fig. 2:**
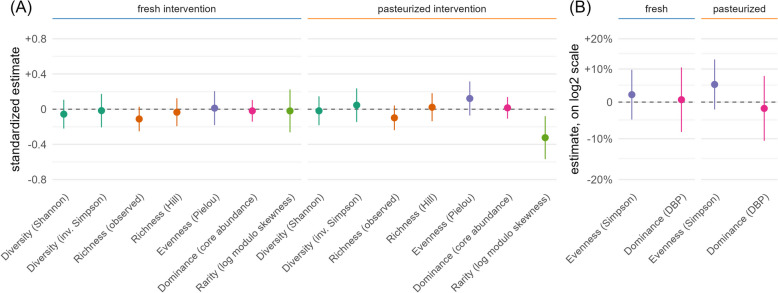
Estimated intervention effects on individual α-diversity measures (grouped by color), with uncorrected 95% confidence intervals. **A** Additive effects based on Normal regression. Estimates are standardized by the respective marker’s standard deviation. The depicted change of 0.2 standard deviations refers to a change of ~ 0.1 (Shannon diversity), ~ 1.9 (inv. Simpson diversity), ~ 8.2 (obs. richness), ~ 2.9 (Hill richness), ~ 0.01 (evenness), ~ 0.02 (dominance), ~ 0.003 (rarity). **B** Multiplicative effects based on log Normal or Gamma regression. No subgroup effect was significant after multiple testing corrections when stratified by baseline diversity

We assessed microbial α-diversity, richness, evenness, and dominance with two indices each (Fig. 2). These measures were mainly equally distributed at baseline when stratified for age, BMI, fiber intake, or sex (Supplementary Table S2), even though α-diversity and richness appeared to be higher in participants older than 50 years. No significant effects were found among the overall and stratified intervention analyses (*q*-values < 0.1). However, a trend toward a decrease in rare species could be observed following the pasteurized sauerkraut intervention (Fig. 2A). Stratification by BMI showed a trend toward higher responsiveness (increased diversity and evenness) to the pasteurized sauerkraut intervention among overweight participants (Supplementary Fig. S3).

#### β-diversity: between-sample variation

**Fig. 3:**
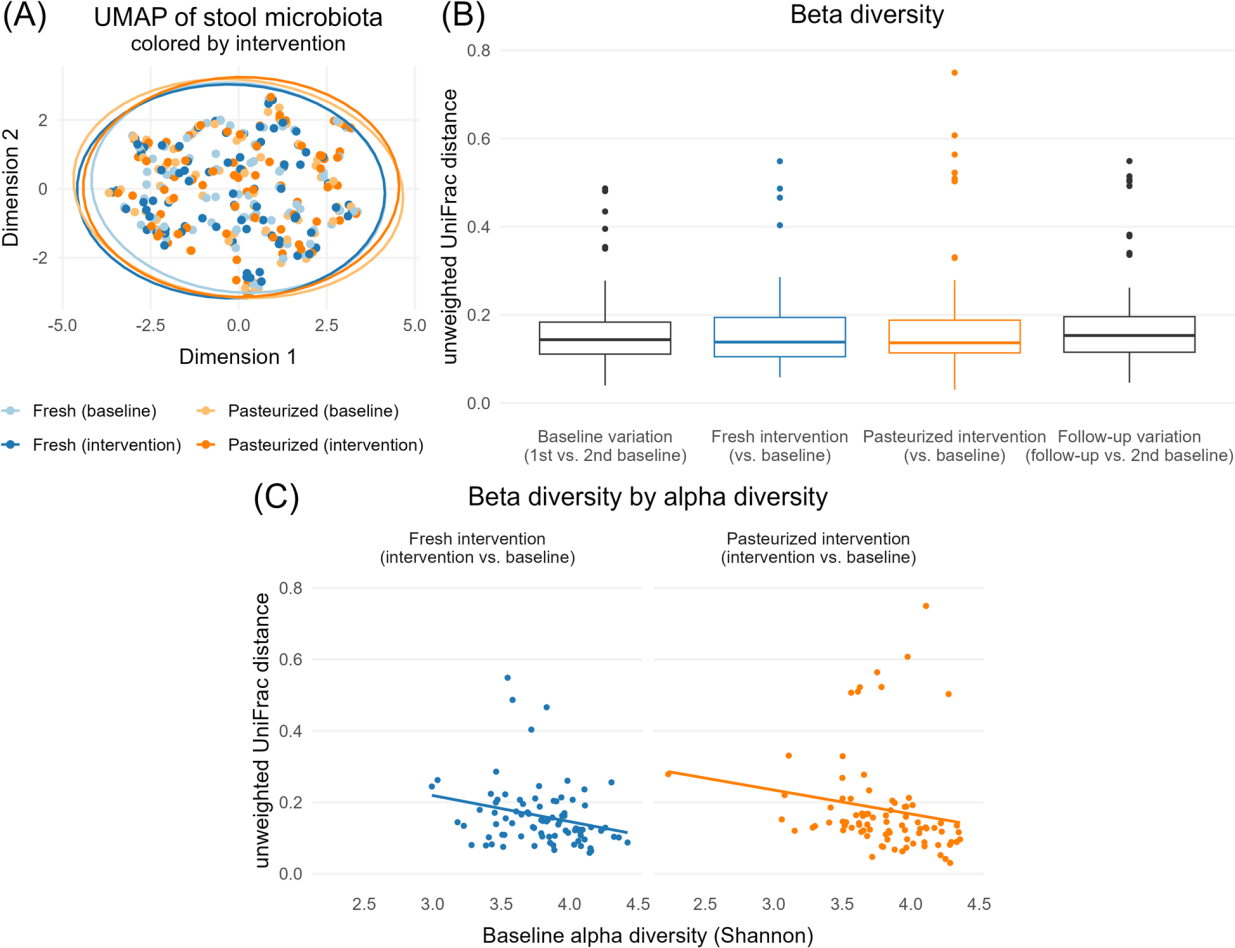
Descriptive changes in microbiota profiles. **A** UMAP representation of individual microbiota profiles before and after the interventions, including ellipses representing the 2D distributions. **B** Unweighted UniFrac distances comparing baseline variation with the observed intervention effects. **C** Correlation of baseline α-diversity (Shannon index) and β-diversity (unweighted UniFrac distance) per intervention

As expected, the intrapersonal β-diversity was overall smaller than interpersonal differences (Supplementary Fig. S4), but the variation in individual gut microbiota profiles throughout our study was rather heterogeneous. The UMAP representation of the observed gut microbiotas—visualizing their overall (dis)similarity on a two-dimensional graph—is depicted in Fig. 3A. Structural associations existed between selected parameters like age, dominant species, or α-diversity with the coordinates in the UMAP representation (Supplementary Fig. S5). However, no structural shift in coordinates was visible when comparing the gut microbiotas before and after either intervention (Fig. 3A). The unweighted UniFrac distances of shared bacteria among samples are depicted in Fig. 3B. The bacterial change induced by either intervention was not more substantial than the baseline variation (e.g., the distances between the individual bacterial profiles after the two washout phases), indicating no global shift in bacterial composition. Similar analyses based on the weighted UniFrac distances or Bray–Curtis dissimilarity lead to the same finding (Supplementary Fig. S6). However, β-diversity differed among study participants. Some participants had highly stable gut microbiotas, with short UMAP coordinate distances between study time points, while others showed more unstable profiles (Supplementary Fig. S7). We wondered whether these differences were related to structural differences in diversity and associated baseline α-diversity with β-diversity to test whether participants with lower baseline diversity would show a greater intervention effect. We found that intrapersonal variation (comparing pre- and post-intervention gut microbiota profiles) showed some degree of a negative correlation with baseline α-diversity (− 0.25 and − 0.17 for the fresh and pasteurized intervention, respectively, see Fig. 3C). However, in contrast to unweighted UniFrac β-diversity, no correlation with baseline α-diversity could be observed for weighted UniFrac or the Bray–Curtis dissimilarity. In other words, the change in phylogenetic richness (unweighted UniFrac considers only the absence or presence of species) was consistently smaller for participants with a higher baseline diversity.

### Effect on species’ relative abundance

**Fig. 4:**
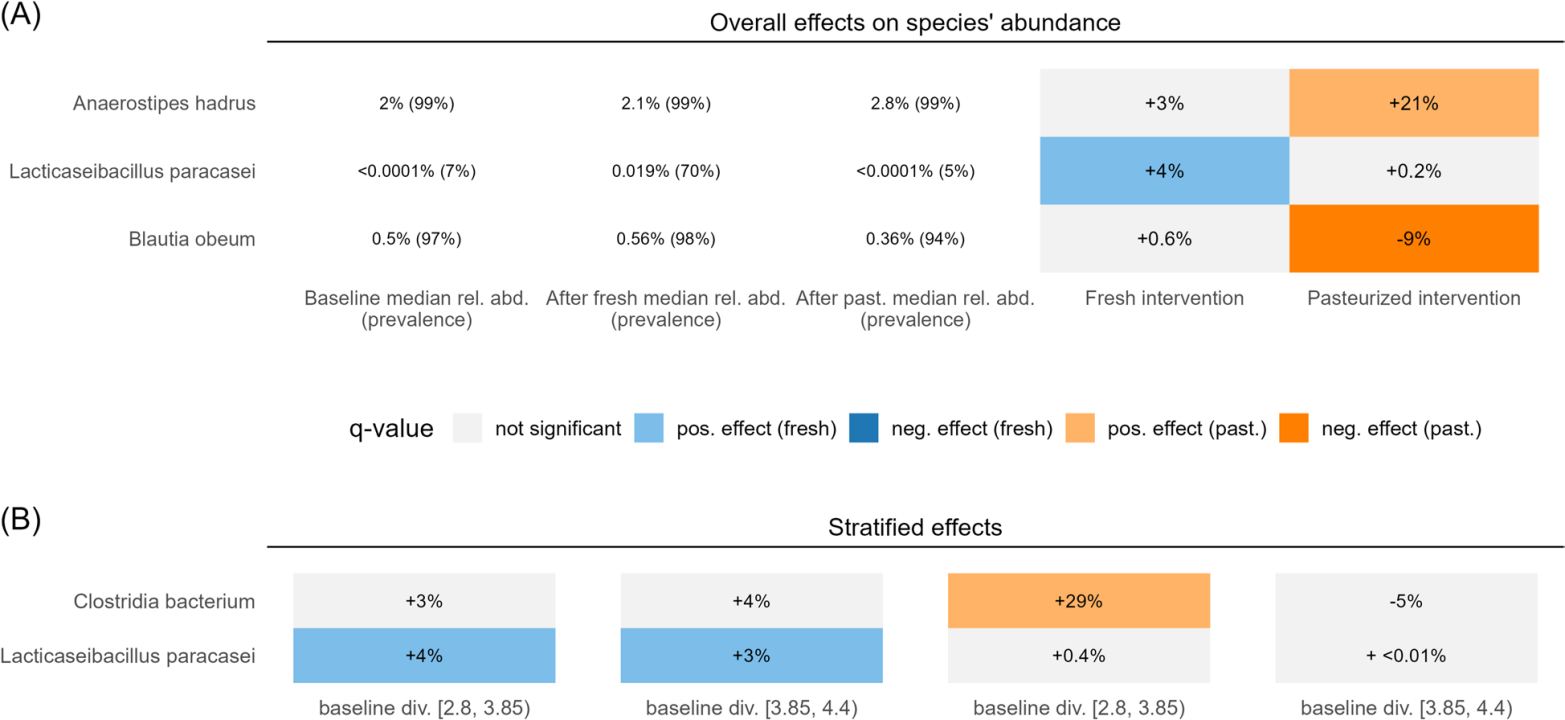
Estimated intervention effects on species’ relative abundance. **A** Species with significant effects in the overall study population (*q*-value < 0.1). **B** Species with significant effects after stratification by baseline Shannon diversity

Phylogenetic differences at the species level do not necessarily translate into differences visible in measures of microbial diversity. Despite the seemingly unaffected microbial profiles analyzed on a broader scale, we identified significant differences in single species’ abundances related to our interventions. As expected, the prevalence and relative abundance of *L. paracasei* in participants' stool increased significantly after the fresh but not the pasteurized intervention phase (Fig. 4A). *Anaerostipes hadrus*, a common gut commensal detected in all study participants, increased significantly in relative abundance during the pasteurized sauerkraut intervention. Furthermore, the relative abundance of *Blautia obeum* was significantly decreased after this intervention. At the species level, individuals with a lower baseline diversity, which had been demonstrated to exhibit slightly higher variability in species abundances, demonstrated a significant increase in *Clostridia *(Fig. 4B).

Because changes in commensal species abundances depend on the baseline microbial composition, we were curious to see if different species might be affected in different strata. Additional affected species were detected in participants older than 50 years, where α-diversity was observed to be higher at baseline (Supplementary Fig. S11 and Table S2). These involved less well-characterized species, of which only *Phocaeicola dorei* has been taxonomically characterized. Furthermore, stratification by BMI revealed additional reactions in overweight individuals (Supplementary Fig. S11). A yet uncharacterized species decreased after the fresh intervention, and *Blautia faecis* markedly increased after consuming the pasteurized sauerkraut. Participants following a low-fiber diet showed an additional increase in *Anaerobutyricum hallii* following pasteurized sauerkraut consumption. Our data revealed no substantial differences between the sexes (Supplementary Fig. S11).

Taken together, the pasteurized sauerkraut was mainly associated with a relative increase in *A. hadrus* and a less pronounced decrease in *B. obeum*, but significant variations in the relative abundance of additional species from the *Lachnospiraceae* family were observed among overweight or older participants and individuals following a low-fiber diet.

### Bacterial metabolites

**Fig. 5:**
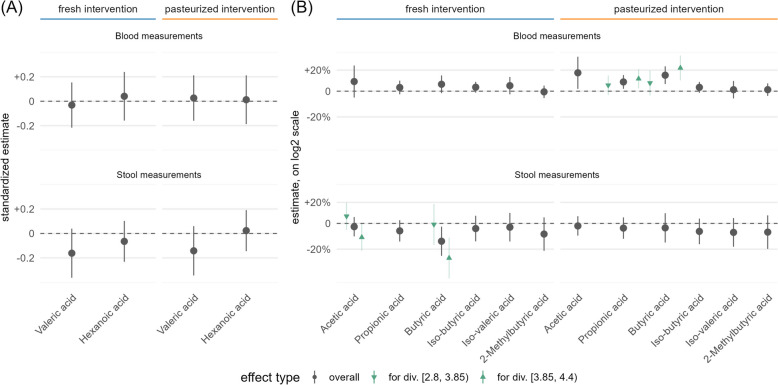
Estimated intervention effects on serum and fecal SCFA levels, including 95% confidence intervals; significant differences depending on baseline Shannon diversity are indicated by green arrows. (**A**) Additive effects based on Normal regression. Estimates were standardized by the respective marker’s standard deviation. The depicted change of 0.1 standard deviations refers to a change of ~ 0.6 ng/ml (valeric acid in the blood), ~ 9.4 µg/g (valeric acid in stool), ~ 2.0 ng/ml (hexanoic acid in the blood), and ~ 12.4 µg/g (hexanoic acid in stool). **B** Multiplicative, exponentiated intervention effects on markers based on log Normal or Gamma regression, depicted on a log2-transformed *y*-axis

We assessed SCFA levels from serum and stool specimens, which were not correlated (Supplementary Fig. S12). In serum, acetic acid, propionic acid, and butyric acid increased significantly only following the pasteurized sauerkraut intervention (Fig. 5). Conspicuously, serum acetic acid increased significantly in overweight participants (Supplementary Fig. S13).

**Fig. 6:**
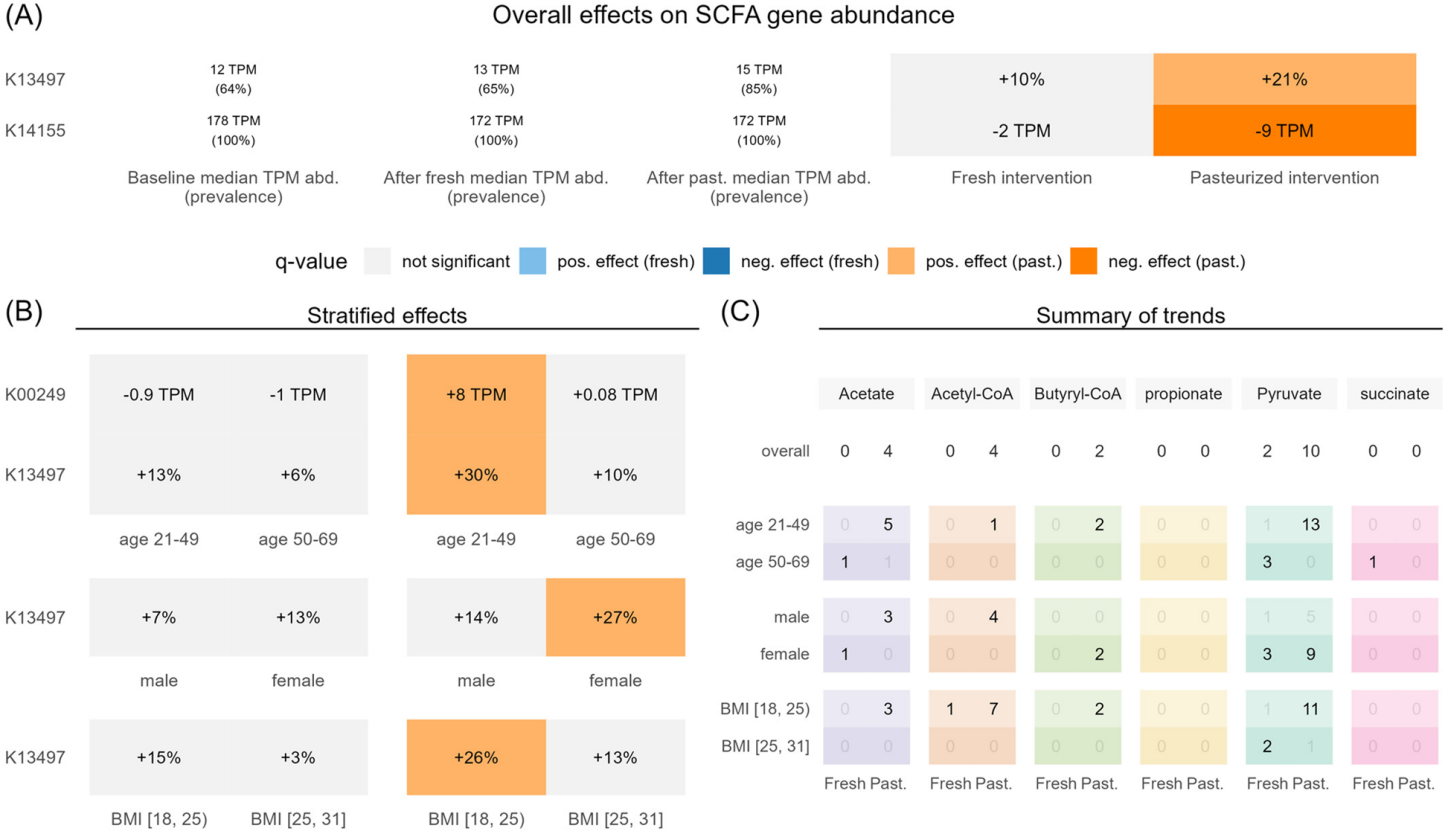
Estimated intervention effects on KEGG orthologue (KO) abundances. **A** Significant effects in the overall study population (*q* value < 0.1). **B** Significant effects after stratification by age, sex, and BMI. **C** Overview of trends of affected KO’s in the overall study population and strata; presented are the numbers of KO’s with changed relative abundance following each intervention (*q*-values > 0.1, *p*-values <0.05), higher numbers compared to their comparative value in each subgroup are highlighted in darker shade

Fecal levels of butyric acid decreased significantly following fresh sauerkraut consumption. Higher fecal SCFA production may be expected in participants who consume more fiber and in those with a more diverse gut microbiota, which may be more efficient in fermentation. It is noteworthy that the observed effects (the decline in fecal SCFA concentration, and the increase in serum SCFA concentration) appeared to be more pronounced in these subgroups (Fig. 6B, Supplementary Figs. S13 and S14).

### Functional analysis

Motivated by the increased serum SCFA levels following pasteurized sauerkraut consumption, we assessed changes in KEGG orthologues (KOs) relevant to SCFA production. Out of the 193 SCFA-related KOs, 89 were identified with a relevant relative abundance (see “Materials and methods” section). In line with our previous findings, only the pasteurized sauerkraut intervention affected SCFA-related KOs significantly (Fig. 6A). Three KOs emerged to be significantly affected following pasteurized sauerkraut consumption. K13497 encodes an enzyme involved in tryptophan synthesis, which produces pyruvate (a precursor of SCFAs), among other products (Fig. 6A, B). Another KO related to pyruvate production during cysteine and methionine metabolism, namely K14155, was observed to occur in reduced abundance following the pasteurized intervention. Next, we wanted to determine the relevance of the bacteria associated with significant changes during the pasteurized sauerkraut intervention for the variation in SCFA-relevant KOs. A relevant correlation between KOs involved in Butyryl-CoA formation, which is a precursor of butyrate, and *A. hadrus* could be observed (Supplementary Fig. S16).

Integrating mapped KO genes in KEGG pathways revealed no significant alterations related to sauerkraut consumption. However, the trends observed after the pasteurized sauerkraut intervention were consistently negative in our study population (Supplementary Fig. S17). The more pronounced trends involved reductions in lipoic acid metabolism, arabinogalactan biosynthesis, and other glycan degradation. Taken together, this data underlines a higher impact of the pasteurized sauerkraut intervention on the microbial functions assessed. However, the changes in single KOs were not expressed in a consistent variation of metabolic, genetic information processing, or cellular processing pathways.

## Discussion

In this randomized crossover trial, we observed changes in single bacterial species’ relative abundance in participants’ feces after consuming fresh and pasteurized sauerkraut daily for 4 weeks. Significant compositional changes in α- and β-diversity could not be found. Interestingly, only the pasteurized sauerkraut was associated with a significant increase in serum SCFA levels.

As expected, LAB were identified as the prominent members of the fresh sauerkraut used in this study. However, the microbial composition, including associated products, possibly differs between the fresh and the pasteurized sauerkraut because they have been produced differently. Spontaneously fermented foods like our pasteurized sauerkraut are expected to contain a more diverse microbiota (before pasteurization) compared to starter culture-produced foods like our fresh sauerkraut [16]. It can be reasonably inferred that *L. paracasei* was the predominant species delivered to our participants during the fresh sauerkraut intervention phase. This is supported by the observation of an increased prevalence of this species in stool samples collected after the intervention period. The species disappeared again after the washout phase, indicating that our study design was appropriate. Similarly, Nielsen et al., in a parallel arm intervention study, reported an increase in fecal sauerkraut-related bacteria only after consumption of fresh but not pasteurized sauerkraut [22]. Even though this seemed to have no impact on overall α-diversity, consumption of both sauerkraut types appeared to impact β-diversity (weighted and unweighted UniFrac distances) in this study with irritable bowel syndrome (IBS) patients. Our results suggest a higher gut microbial resilience in healthy individuals, as we did not find marked changes in microbial α- and β-diversity induced by the consumption of either sauerkraut. UMAP representation revealed a heterogeneous variation of microbial profiles. Generally, a more diverse microbial community is expected to be more stable over time. Chen et al. compared the change in microbial composition over 4 years and observed a decreased Bray–Curtis dissimilarity when the baseline Shannon diversity was higher [59]. In a cross-over trial involving 14 obese men with metabolic syndrome, responders to dietary interventions (fiber supplementation and weight loss) were characterized by a lower baseline inverse Simpson diversity [60]. IBS is often associated with reduced microbial diversity, potentially explaining their responsiveness to sauerkraut observed by Nielsen et al. [22, 61]. Thus, we hypothesized that a greater diversity at baseline would result in a more stable bacterial profile that is less susceptible to environmental factors, including our interventions. We found no correlation between baseline Shannon diversity and Bray–Curtis dissimilarities but between the former and unweighted UniFrac distances, shifting the focus to the number of species present at baseline. Higher baseline species richness has been observed to be a determining factor for gut microbial stability in response to a high-fiber diet in another trial with 19 healthy participants [62]. However, we could not identify a common microbial shift in response to sauerkraut in participants with a smaller baseline diversity. The relation between microbial diversity and microbiome stability is indeed not yet clearly established. Importantly, using diversity metrics that condense data into a single value to describe microbial diversity may not capture all the nuances of microbiome stability [63].

In addition, dietary diversity has been identified as a driver of microbial diversity [20]. Thus, increased consumption of various fermented foods, as in the study by Wastyk et al., who observed an increase in Shannon diversity, maybe a more promising approach to influencing microbial diversity in healthy people. In a large-scale cross-sectional study comparing the microbial profiles of general fermented food consumers and non-consumers from the American Gut Project cohort, Taylor and colleagues found no difference in the fecal microbial α-diversity but slight phylogenetic differences in the microbial communities of the two groups [14]. They associated regular consumption of fermented foods with fermented food-derived but also unrelated bacteria in the gut [14]. Even though we found no effect on the specific species identified in this study, we associated *A. hadrus* with the consumption of the used pasteurized sauerkraut, which is a known human gut commensal [64]. This species has been observed to be common in higher abundance in healthy individuals (compared to patients with inflammatory bowel disease) and may be an important member of the intestinal cross-feeding network as it extracellularly degrades prebiotic fructooligosaccharides (FOS) [65, 66]. Furthermore, we found a relative decrease in *Blautia obeum* (formerly known as *Ruminococcus obeum* [67]), another common gut commensal [68]. *Clostridia*, which increased in participants with lower baseline diversity has been ascribed a central role in gut homeostasis and gut microbiota stability [69]. The species affected in our study differed according to BMI, age, fiber intake, and baseline diversity, suggesting that intestinal responses go beyond the introduction of digested species but rather affect species present in the community, as suggested by Wastyk and colleagues [9]. However, the hypothesis that the gut microbiota is indirectly affected by the consumption of sauerkraut needs further verification, as we have no reliable information on the microbial composition (or the remaining microbial components) of the pasteurized sauerkraut itself. Notably, the species associated with the pasteurized sauerkraut in different strata all belonged to the *Lachnospiracea*e family comprising many important SCFA producers of the human gut [70].

We assessed SCFA levels in serum and stool. Previous studies have suggested no direct correlation between serum and fecal SCFA levels, as both lack the proportion consumed by host cells [71, 72]. SCFA concentration assessment in stool quantifies microbial production’s remains after host absorption, while serum levels represent the fraction of SCFAs reaching peripheral tissue. A large proportion is readily consumed by enterocytes or liver cells before reaching circulation [73]. While fecal SCFA levels tended to decrease following fresh sauerkraut consumption, an increase could be observed in serum following the pasteurized sauerkraut intervention. The lack of increased SCFA in feces might be attributable to increased uptake by the host. In healthy conditions, the presence of SCFAs upregulates the expression of mucosal SCFA transporters, enhancing their absorption alongside passive diffusion across the intestinal barrier [74]. This is supported by the observation that the fecal decrease of SCFA was more pronounced in participants consuming more fiber. Increased fecal SCFA levels have furthermore been associated with diseases such as IBS or obesity [75, 76]. Serum SCFA levels, on the other hand, are of physiological relevance, and circulating SCFAs have been considered valuable biomarkers for metabolic health [71]. BCFAs, resulting mainly from protein fermentation, which have been correlated with negative health implications, were unaffected [77]. It is possible that the SCFAs were contained in the sauerkraut and that they reached the circulation through the upper intestine [78]. Indeed, sauerkraut contains relevant amounts of SCFAs, especially acetic acid [78]. The fresh sauerkraut was slightly more acidic than the pasteurized sauerkraut, probably due to the activity of *L. paracasei*, which produces large amounts of L( +)-lactic acid. However, we analyzed 12-h fasting serum SCFA levels, and orally delivered SCFAs are expected to disappear after a few hours [72, 78]. Applying the hypothesis test for theIn line with our previous results, this analysis showed significant changes only during the pasteurized sauerkraut intervention. However, these changes were not reflected in KEGG metabolic pathways, which were only weakly affected. Consistent with our previous findings, functional changes were limited to specific genes rather than community-wide adaptations.

This study had several strengths and limitations. A significant limitation is the uncertainty about the composition of the pasteurized sauerkraut microbiota and its potential difference from the fresh sauerkraut. Still, we could link pasteurized sauerkraut consumption to specific bacterial species in human stool and thereby analyze its physiological effects. In addition, we had no control over whether participants ate the sauerkraut every day at home. However, compliance assessed by questionnaires was high. Another disadvantage of this dependency on volunteers is the characteristic interest in healthy nutrition and a preference for the taste of sauerkraut in our study population. Furthermore, the stool provides non-invasive but limited insight into the gut microbial community. Assessment of SCFA production, in particular, may be complex as fermentation is most active in the proximal colon and decreases towards the distal gut [26]. In addition, the analysis of self-collected stool specimens involves some difficulties. Due to individual variations in normal defecation frequency, stool specimen storage at home and preservation quality were occasionally dissimilar. This, as well as the site of collection, has been shown to impact the microbial composition of the donated specimen [79]. However, we have tried to minimize these downsides of fecal microbiome analysis, which is standard practice, with continuous cooling and stabilization in ethanol (for sequencing). Participants determined their stool type based on the Bristol Stool Form Scale. Thus, we cannot rule out misclassification, mainly because we asked participants to indicate their average stool consistency during the last week of each study phase. Still, this medical tool is based on well-described and illustrated categories, minimizing classification issues. Indeed, the Bristol Stool Form Scale is no perfect measure of overall intestinal transit time. Nonetheless, the correlation has been established as meaningful [39]. Furthermore, pH was evaluated to the nearest 0.5 (stool) or 0.3 (sauerkraut), potentially ignoring more minor fluctuations.

A major strength of this study is its crossover study design, in which each participant serves as their control. In addition, it effectively doubles the population size since each participant underwent both interventions. Compared to previous intervention trials with fermented food consumption, this study comprises, by far, the largest sample size [9, 15, 22, 80].

In summary, our results suggest that microbial changes occurred on the species level. The pasteurized sauerkraut appeared to induce more pronounced effects on the fecal microbiome composition and metabolic function. At the same time, the main finding related to the fresh intervention was the recovery of sauerkraut-derived bacteria in human stool. As the substrate and, thereby, the fiber content of our interventions was similar, the pasteurized sauerkraut microbes themselves or their products might be responsible for our findings. Non-viable microorganisms in fermented foods might confer physiological effects, as reflected in the “postbiotic” concept. Postbiotics are a “preparation of inanimate microorganisms and/or their components that confers a health benefit on the host” [31].

## Conclusion

We conclude that fresh and pasteurized sauerkraut had no overall impact on the gut microbiota of healthy participants, even though single species changed significantly in relative abundance. However, the significant increase in serum SCFA levels during this intervention might link sauerkraut consumption with a potential impact on systemic health. The specific composition of the sauerkraut might be an important determinant of the gut microbiota-host interaction.

## Supplementary Information

Supplementary Material 1: Figure S6. Species composition of the fresh sauerkraut used in our study, for the three sampled sauerkraut glasses. Glass 1 has been stored for seven weeks before sampling. Species with less than 0.1% relative abundance in all samples are categorized as “Others”. Figure S7: Distributions of stool specimen characteristics in the overall study population and stratified for sex, age, and BMI; (A) Bristol Stool Form (“stool type”) the week before any stool collection; (B) pH value of the donated stools; (C) mean defecation frequency the week before stool donation. Linear mixed regression model (similar to the models reported in the main manuscript) on these stool specimen characteristics did not reveal any relevant overall or stratified intervention effects. Figure S8: Estimated intervention effects on individual α-diversity measures (grouped by color) stratified by (from top to bottom) baseline Shannon diversity, age, sex, BMI, and daily fiber intake. Effects are shown accompanied by 95% confidence intervals which are uncorrected for multiple testing and should thus be interpreted with some caution. No effect was significant after correction for multiple testing. Figure S9: Comparison of intra- and inter-personal β-diversity, based on unweighted and weighted UniFrac distances and Bray–Curtis dissimilarities. Figure S10: UMAP representation colored by dominant species, sex (including ellipses representing the 2D distributions), age, baseline BMI and Shannon diversity. Figure S11: β-diversity at baseline and comparing pre- and post-intervention measurements, based on weighted UniFrac distances (left) and Bray–Curtis dissimilarities (right). Figure S12: UMAP representation of microbiota profiles at each study time point. Individual microbial profiles are connected by polygons highlighting differences in microbial stability. Figure S13: Estimated control variable effects on individual α-diversity measures (grouped by color), with uncorrected 95% confidence intervals which should be interpreted with some caution. Top plot: additive effects based on Normal regression. Estimates are standardized by the respective marker’s standard deviation. The depicted change of 0.2 standard deviations refers to a change of ~ 0.1 (Shannon diversity), ~ 1.9 (inv. Simpson diversity), ~ 8.2 (obs. richness), ~ 2.9 (Hill richness), ~ 0.01 (evenness), ~ 0.02 (dominance), ~ 0.003 (rarity); bottom plot: multiplicative effects based on log Normal or Gamma regression. Figure S14: Estimated control variable effects on blood serum SCFA levels, including uncorrected 95% confidence intervals; top plot: additive effects based on Normal regression. Estimates were standardized by the respective marker’s standard deviation. The depicted change of 0.1 standard deviations refers to a change of ~ 0.6 ng/ml (valeric acid) and ~ 2.0 ng/ml (hexanoic acid); bottom plot: multiplicative, exponentiated intervention effects on markers based on log Normal or Gamma regression, depicted on a log2-transformed y-axis. Figure S15: Estimated control variable effects on fecal SCFA levels, including uncorrected 95% confidence intervals; top plot: additive effects based on Normal regression. Estimates were standardized by the respective marker’s standard deviation. The depicted change of 0.1 standard deviations refers to a change of ~ 9.4 µg/g (valeric acid) and ~ 12.4 µg/g (hexanoic acid); bottom plot: multiplicative, exponentiated intervention effects on markers based on log Normal or Gamma regression, depicted on a log2-transformed y-axis. Figure S16: Estimated intervention effects on species’ relative abundance following stratification by age, sex, BMI, and daily fiber intake (q value < 0.1). Figure S17: Correlation of SCFA levels in serum and stool. Figure S18: Estimated intervention effects on serum SCFA level stratified by (from top to bottom) baseline Shannon diversity, age, sex, BMI, and daily fiber intake. Effects are shown accompanied by 95% confidence intervals which are uncorrected for multiple testing and should thus be interpreted with some caution. Figure S19: Estimated intervention effects on fecal SCFA level stratified by (from top to bottom) baseline Shannon diversity, age, sex, BMI, and daily fiber intake. Effects are shown accompanied by 95% confidence intervals which are uncorrected for multiple testing and should thus be interpreted with some caution. Figure S20: Estimated intervention effects on the relative abundance of SCFA-related KOs following stratification by age, sex, BMI, and daily fiber intake (q value < 0.1). Figure S21: Correlation of species that changed significantly during an intervention in this study and KEGG orthologues (KOs) relevant for SCFA metabolism. KOs are grouped by their association with specific products. Figure S22: Estimated intervention effects on KEGG pathways’ relative abundance. Effects highlighted in orange had p-values < 0.05 (not corrected for multiple testing) which display trends but should be interpreted with some caution. Pathway group abbreviations: “CP” = Cellular Processes, “GIP” = Genetic Information Processing. Table S3: Comparison of amplified DNA present in the used sauerkraut to identify present species; the table presents the mean reads from three samples taken from the same glass. Fresh glass 1 had been stored for 7 weeks before sampling. Table S4: Baseline characteristics, including α-diversity measures, in stratification groups. Presented are mean values for metric characteristics and relative frequencies for categorical characteristics assessed before starting the first intervention phase.The subgroup analysis “baselineDiv “ is based on baseline Shannon diversity.

## Data Availability

The complete data and code for this study are available at https://github.com/bauer-alex/IPE_sauerkrautTaxonomy.

## Abbreviations

BCFAs: Branched-chain fatty acids
CPM: Copies per million
FFQ: Food-frequency questionnaire
GLMM: Generalized linear mixed regression model
IGC: Integrated gene catalog
KO: KEGG orthologue
LAB: Lactic acid-producing bacteria
SCFAs: Short-chain fatty acids
TPM: Transcripsper million
UHPLC/MS/MS: Ultra-high-performance liquid chromatography/tandem accurate mass spectrometry
UMAP: Uniform Manifold Approximation and Projection
