# Supplementary material for: The impact of regular sauerkraut consumption on the human gut microbiota: a crossover intervention trial
Source: Microbiome. 2025 Feb 12;13:52. doi: 10.1186/s40168-024-02016-3 (PMC11817299; doi:10.1186/s40168-024-02016-3)
Supplement: Supplementary file 2 — Supplementary Material 1: Figure S6. Species composition of the fresh sauerkraut used in our study, for the three sampled sauerkraut glasses. Glass 1 has been stored for seven weeks before sampling. Species with less than 0.1% relative abundance in all samples are categorized as “Others”. Figure S7: Distributions of stool specimen characteristics in the overall study population and stratified for sex, age, and BMI; (A) Bristol Stool Form (“stool type”) the week before any stool collection; (B) pH value of the donated stools; (C) mean defecation frequency the week before stool donation. Linear mixed regression model (similar to the models reported in the main manuscript) on these stool specimen characteristics did not reveal any relevant overall or stratified intervention effects. Figure S8: Estimated intervention effects on individual α-diversity measures (grouped by color) stratified by (from top to bottom) baseline Shannon diversity, age, sex, BMI, and daily fiber intake. Effects are shown accompanied by 95% confidence intervals which are uncorrected for multiple testing and should thus be interpreted with some caution. No effect was significant after correction for multiple testing. Figure S9: Comparison of intra- and inter-personal β-diversity, based on unweighted and weighted UniFrac distances and Bray–Curtis dissimilarities. Figure S10: UMAP representation colored by dominant species, sex (including ellipses representing the 2D distributions), age, baseline BMI and Shannon diversity. Figure S11: β-diversity at baseline and comparing pre- and post-intervention measurements, based on weighted UniFrac distances (left) and Bray–Curtis dissimilarities (right). Figure S12: UMAP representation of microbiota profiles at each study time point. Individual microbial profiles are connected by polygons highlighting differences in microbial stability. Figure S13: Estimated control variable effects on individual α-diversity measures (grouped by color), with uncorrected 95% c [file 40168_2024_2016_MOESM1_ESM.docx]

**The Impact of Regular Sauerkraut Consumption on the Human Microbiota: a Crossover Intervention Trial**

**Nelly Schropp ^1^, Alexander Bauer ^1^, Virginie Stanislas ^1^, Agata Anna Bielecka ^2^, Till-Robin Lesker ^2^ Kun D. Huang ^2^, Till Strowig ^2,3^ and Karin B. Michels** ^1^**^,^***

^1^ Institute for Prevention and Cancer Epidemiology, Faculty of Medicine and Medical Centre, University of Freiburg, 79110 Freiburg, Germany;

^2^ Department of Microbial Immune Regulation, Helmholtz Center for Infection Research, 38124 Braunschweig, Germany;

^3^ Center for Individualized Infection Medicine (CiiM), a joint venture between the Helmholtz Center for Infection Research (HZI) and the Hannover Medical School (MHH), 30625 Hannover, Germany

***** Correspondence: tumorepidemiologie@uniklinik-freiburg.de


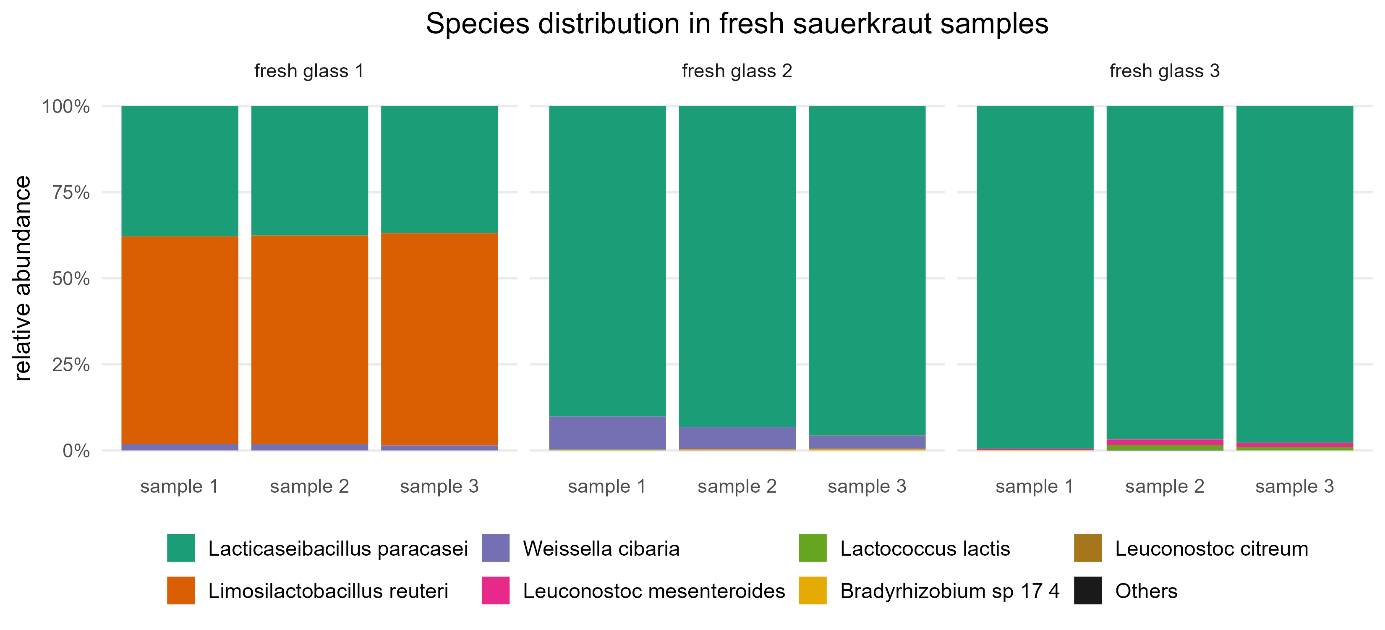


Figure S1: Species composition of the fresh sauerkraut used in our study, for the three sampled sauerkraut glasses. Glass 1 has been stored for seven weeks before sampling. Species with less than 0.1% relative abundance in all samples are categorized as “Others”.

Table S1: Comparison of amplified DNA present in the used sauerkraut to identify present species; the table presents the mean reads from three samples taken from the same glass. Fresh glass 1 had been stored for 7 weeks before sampling.

|  | mean number of estimated reads mapped to known clades | | |
| --- | --- | --- | --- |
|  | glass 1 | glass 2 | glass 3 |
| fresh sauerkraut | 33,729,390. 67 | 486,882.33 | 349,524.67 |
| pasteurized sauerkraut | 1,211 | 1,658.3 | 3,424 |


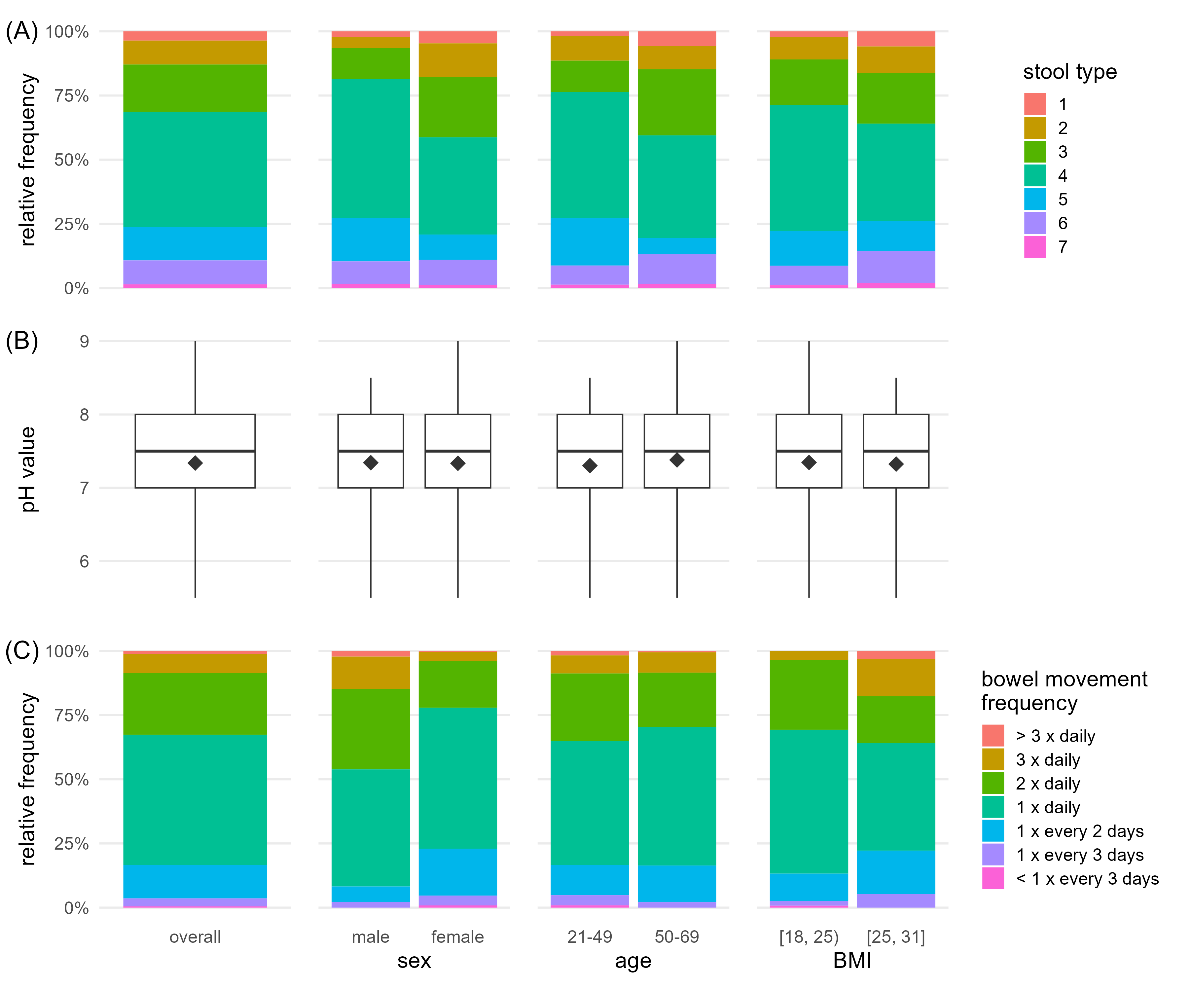


Figure S2: Distributions of stool specimen characteristics in the overall study population and stratified for sex, age, and BMI; (A) Bristol Stool Form (“stool type”) the week before any stool collection; (B) pH value of the donated stools; (C) mean defecation frequency the week before stool donation. Linear mixed regression model (similar to the models reported in the main manuscript) on these stool specimen characteristics did not reveal any relevant overall or stratified intervention effects.

Table S2: Baseline characteristics, including α-diversity measures, in stratification groups. Presented are mean values for metric characteristics and relative frequencies for categorical characteristics assessed before starting the first intervention phase.The subgroup analysis “baselineDiv“ is based on baseline Shannon diversity.

| subgroup | Diversity (Shannon) | age | sex male | BMI | Fiber  intake  [g/day] | Diversity  (inv. Simpson) | Richness (observed) | Richness  (Hill) | Evenness (Pielou) | Evenness  (Simpson) | Dominance  (DBP) | Dominance  (core abd.) | Rarity  (log-md. skewness) |
| --- | --- | --- | --- | --- | --- | --- | --- | --- | --- | --- | --- | --- | --- |
| baselineDiv  <3.85 | 3.58 | 43 | 47% | 24.4 | 23.73 | 21.1 | 149 | 37.0 | 0.72 | 0.15 | 0.14 | 0.83 | 2.05 |
| baselineDiv  ≥3.85 | 4.04 | 46 | 40% | 23.8 | 23.94 | 33 | 199 | 57.7 | 0.76 | 0.17 | 0.10 | 0.75 | 2.05 |
| age < 50 | 3.77 | 32 | 48% | 23.6 | 21.99 | 24.5 | 163 | 43.2 | 0.74 | 0.16 | 0.11 | 0.85 | 2.05 |
| age >= 50 | 3.88 | 57 | 38% | 24.5 | 26.10 | 29.0 | 191 | 48.6 | 0.75 | 0.16 | 0.10 | 0.77 | 2.05 |
| sex male | 3.83 | 38 | 100% | 24.9 | 26.26 | 26.1 | 158 | 46.1 | 0.75 | 0.16 | 0.10 | 0.80 | 2.05 |
| sex female | 3.87 | 49 | 0% | 22.7 | 21.95 | 25.6 | 181 | 48.1 | 0.75 | 0.16 | 0.10 | 0.80 | 2.05 |
| BMI < 25 | 3.83 | 40 | 38% | 22.2 | 23.81 | 26.3 | 178 | 46.1 | 0.75 | 0.16 | 0.10 | 0.81 | 2.05 |
| BMI >= 25 | 3.84 | 50 | 53% | 27.1 | 23.81 | 24.5 | 172 | 46.3 | 0.74 | 0.15 | 0.10 | 0.79 | 2.05 |
| Fiber < 30g | 3.81 | 43 | 42% | 24.2 | 20.72 | 26.8 | 175 | 47.3 | 0.74 | 0.15 | 0.12 | 0.79 | 2.05 |
| Fiber ≥ 30g | 3.75 | 49 | 53% | 23.8 | 38.77 | 26.6 | 162 | 45.3 | 0.74 | 0.16 | 0.12 | 0.80 | 2.05 |


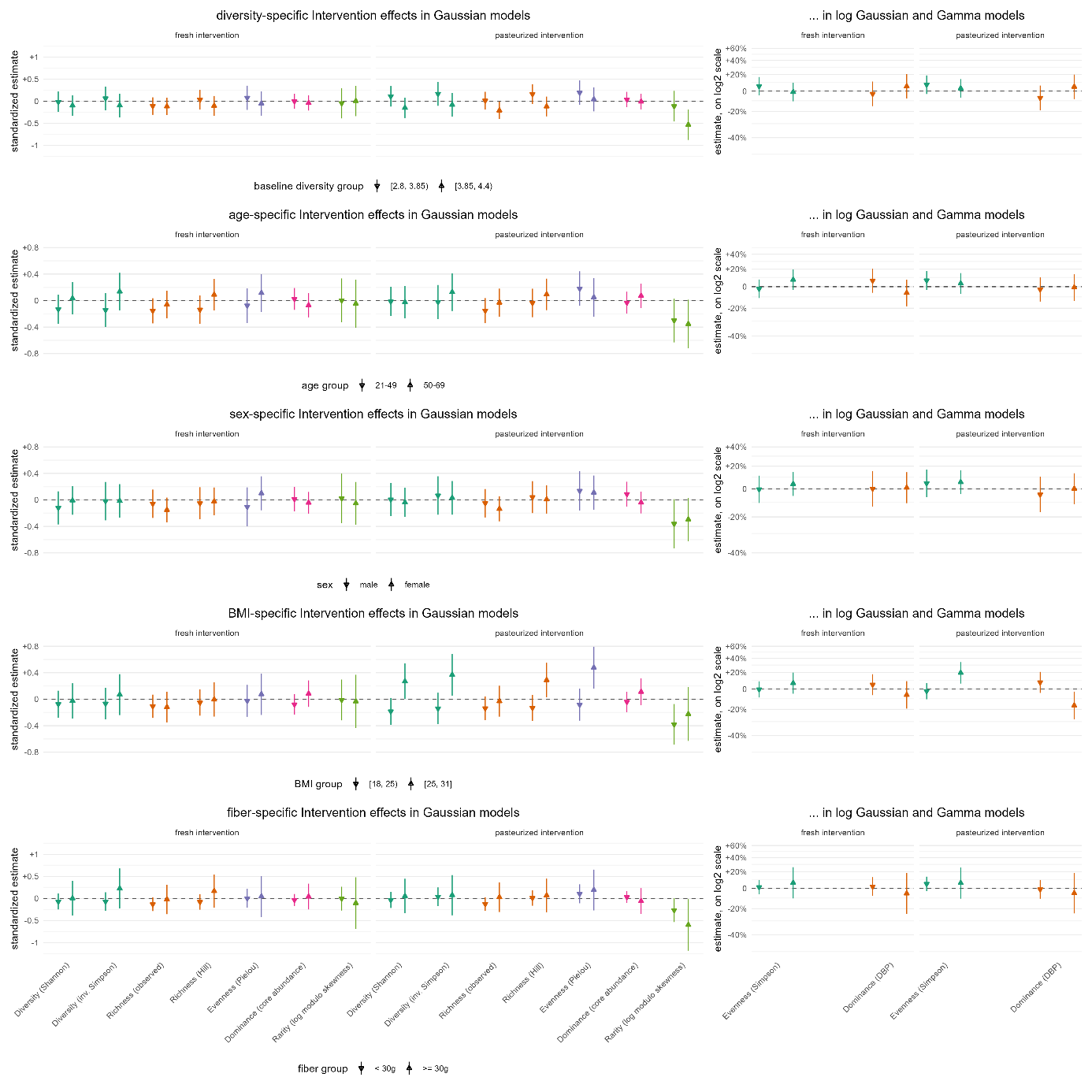


Figure S3: Estimated intervention effects on individual α-diversity measures (grouped by color) stratified by (from top to bottom) baseline Shannon diversity, age, sex, BMI, and daily fiber intake. Effects are shown accompanied by 95% confidence intervals which are uncorrected for multiple testing and should thus be interpreted with some caution. No effect was significant after correction for multiple testing.


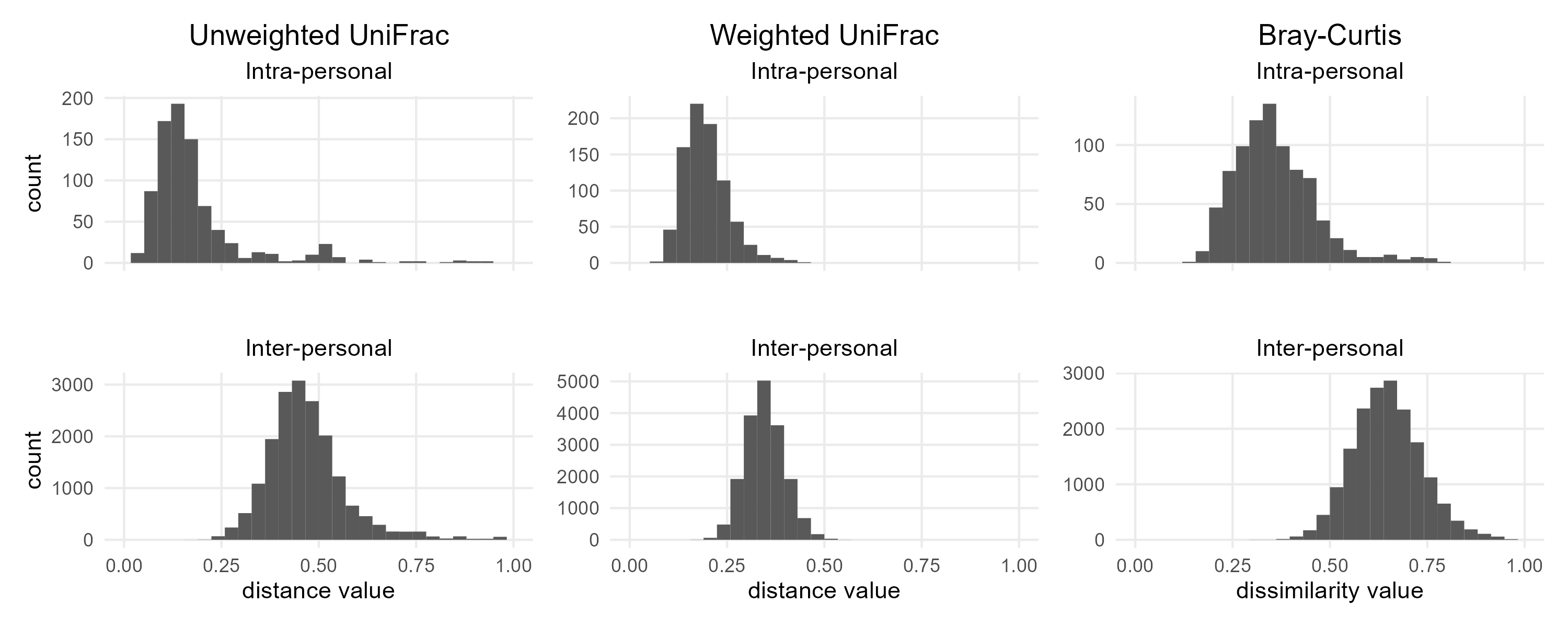


Figure S4: Comparison of intra- and inter-personal β-diversity, based on unweighted and weighted UniFrac distances and Bray-Curtis dissimilarities.


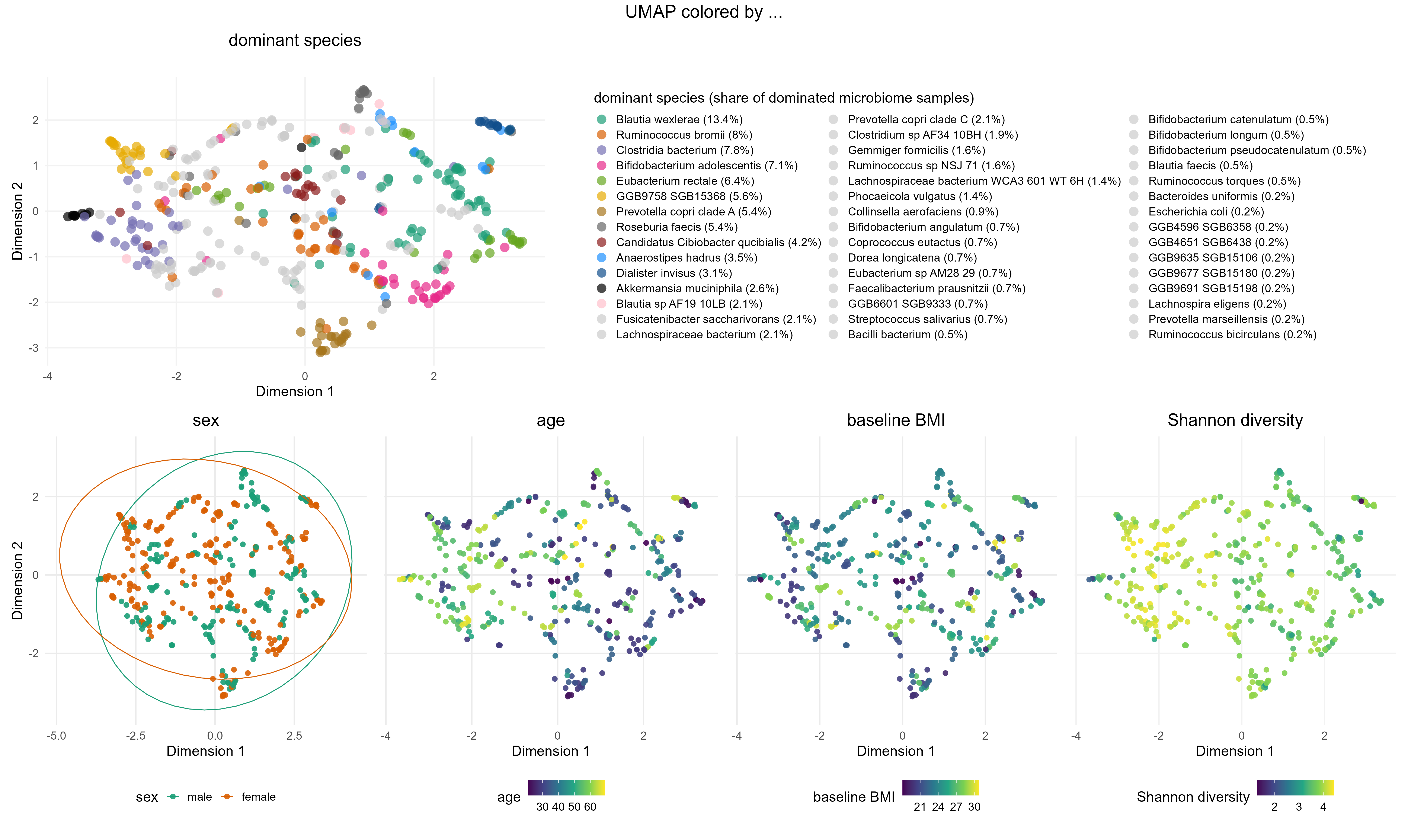


Figure S5: UMAP representation colored by dominant species, sex (including ellipses representing the 2D distributions), age, baseline BMI and Shannon diversity.


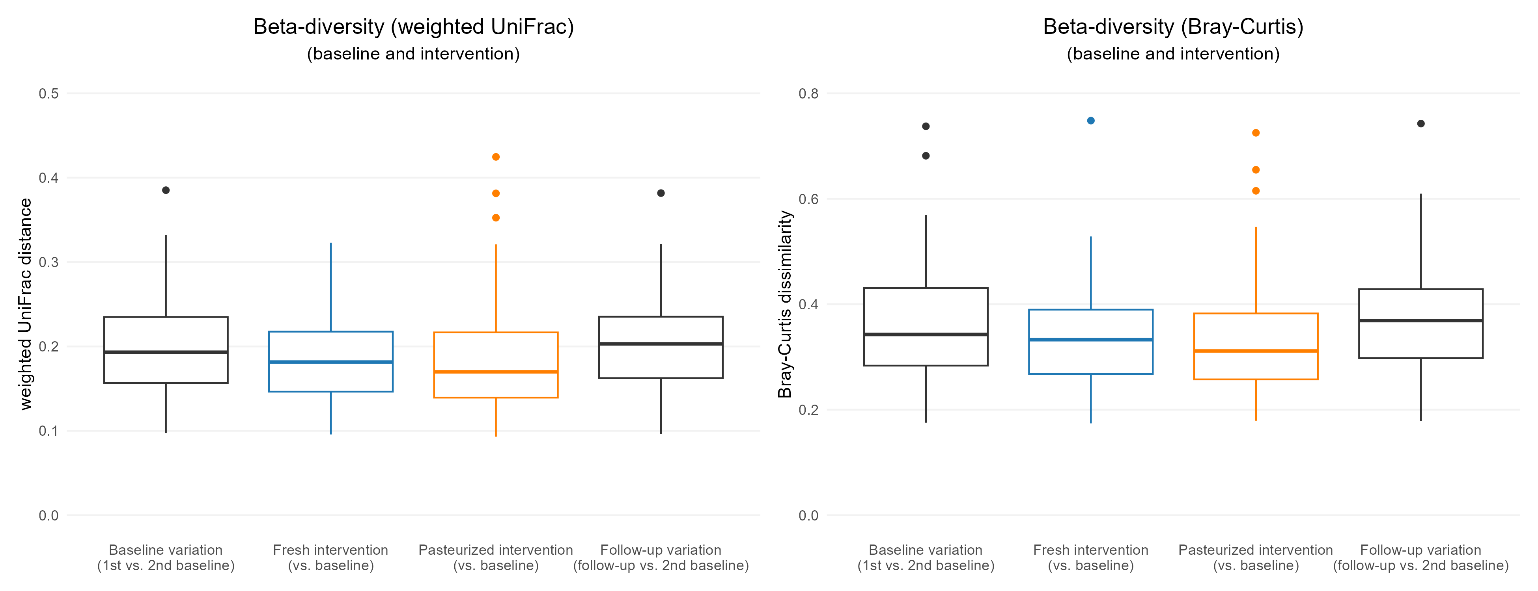


Figure S6: β-diversity at baseline and comparing pre- and post-intervention measurements, based on weighted UniFrac distances (left) and Bray-Curtis dissimilarities (right).


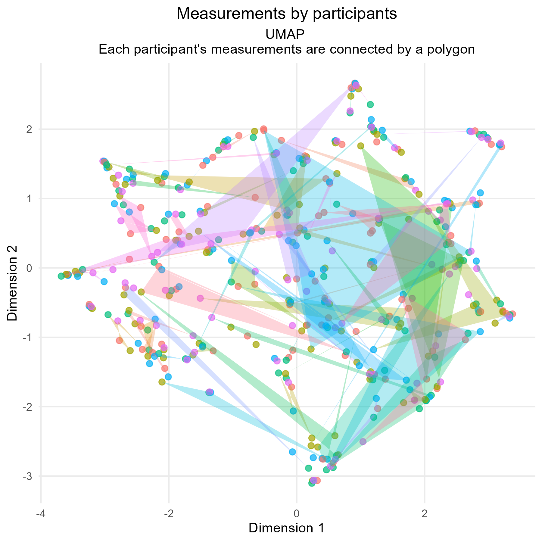


Figure S7: UMAP representation of microbiota profiles at each study time point. Individual microbial profiles are connected by polygons highlighting differences in microbial stability.


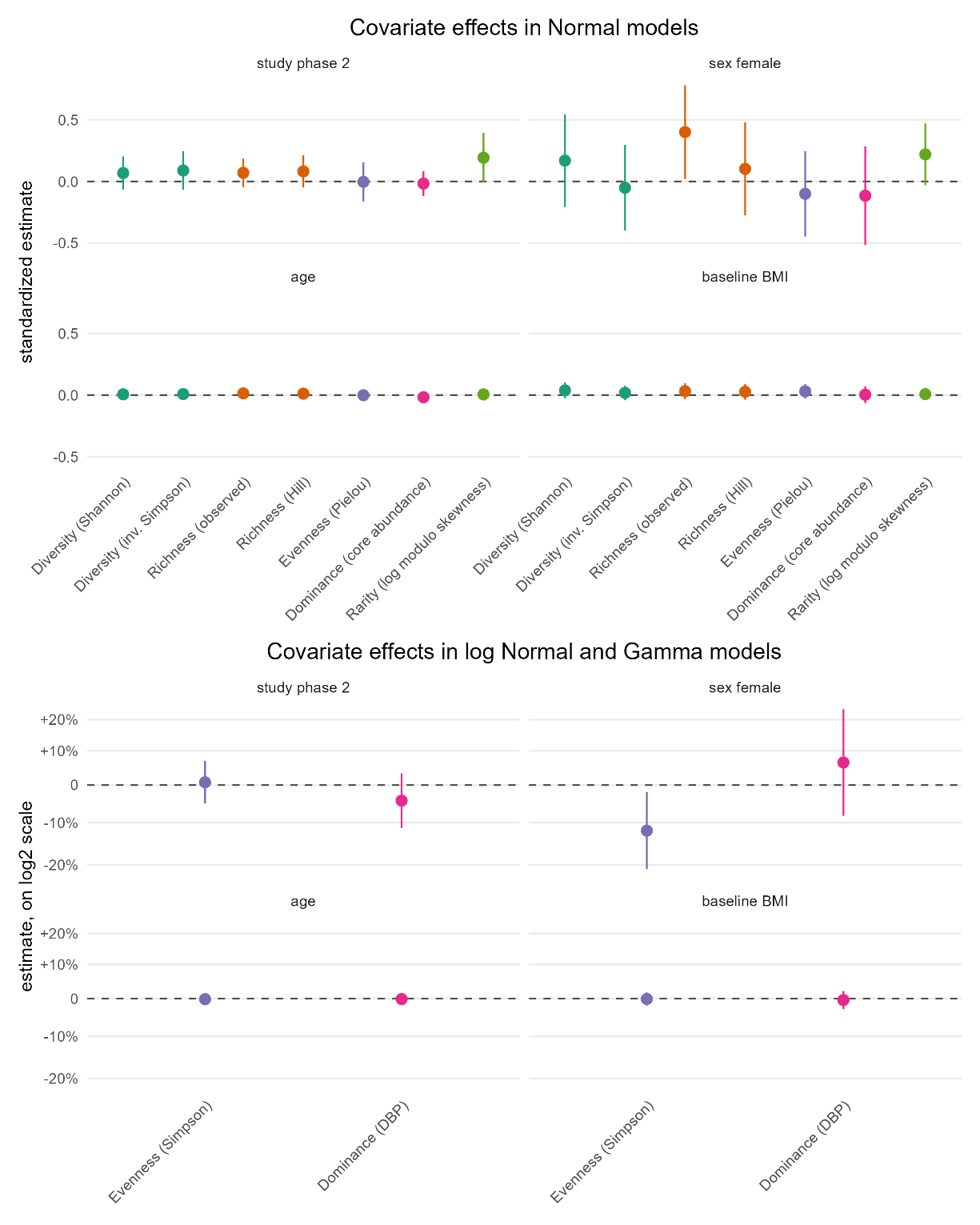


Figure S8: Estimated control variable effects on individual α-diversity measures (grouped by color), with uncorrected 95% confidence intervals which should be interpreted with some caution. Top plot: additive effects based on Normal regression. Estimates are standardized by the respective marker’s standard deviation. The depicted change of 0.2 standard deviations refers to a change of ~0.1 (Shannon diversity), ~1.9 (inv. Simpson diversity), ~8.2 (obs. richness), ~2.9 (Hill richness), ~0.01 (evenness), ~0.02 (dominance), ~0.003 (rarity); bottom plot: multiplicative effects based on log Normal or Gamma regression.


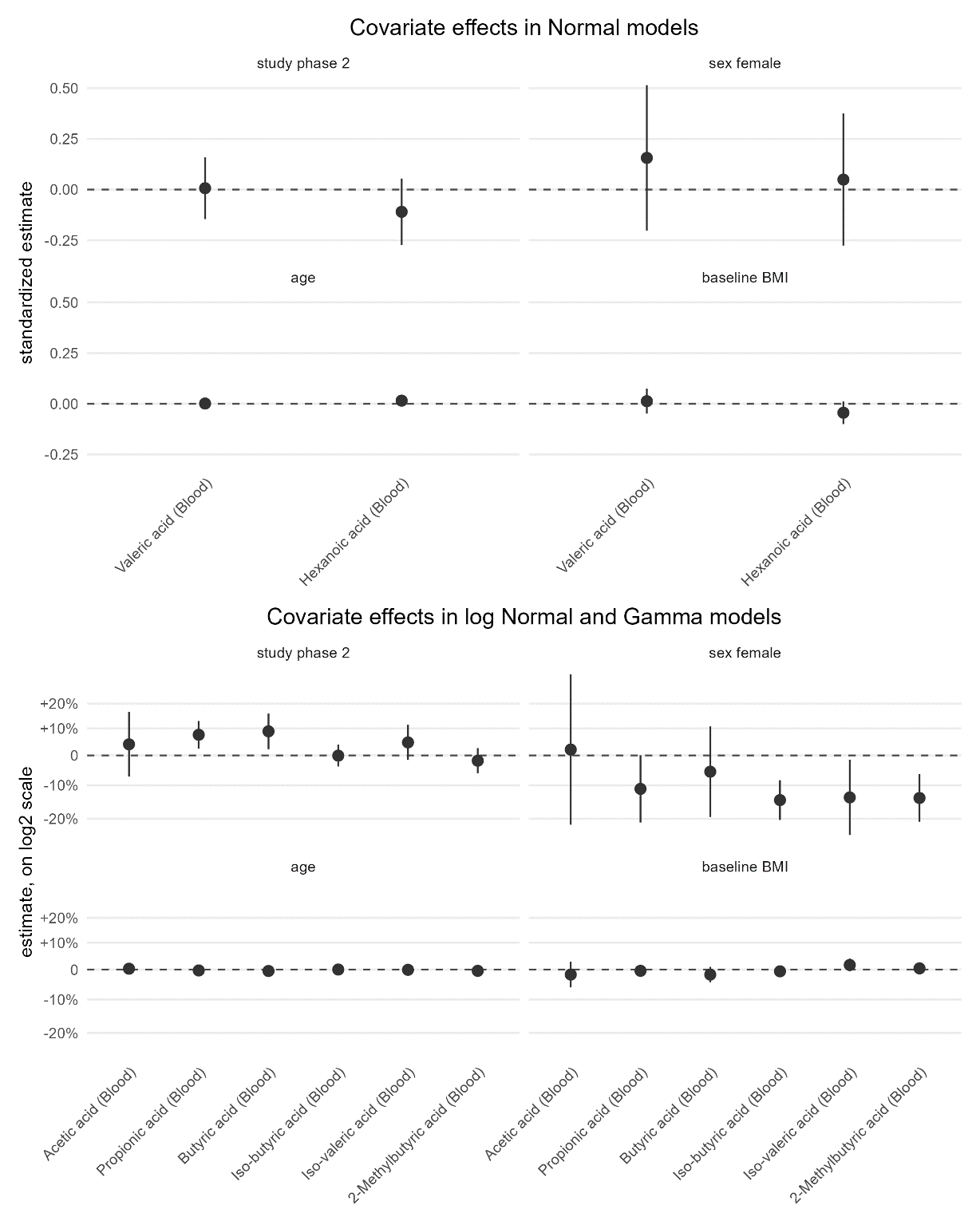


Figure S9: Estimated control variable effects on blood serum SCFA levels, including uncorrected 95% confidence intervals; top plot: additive effects based on Normal regression. Estimates were standardized by the respective marker’s standard deviation. The depicted change of 0.1 standard deviations refers to a change of ~0.6 ng/ml (valeric acid) and ~2.0 ng/ml (hexanoic acid); bottom plot: multiplicative, exponentiated intervention effects on markers based on log Normal or Gamma regression, depicted on a log2-transformed y-axis.


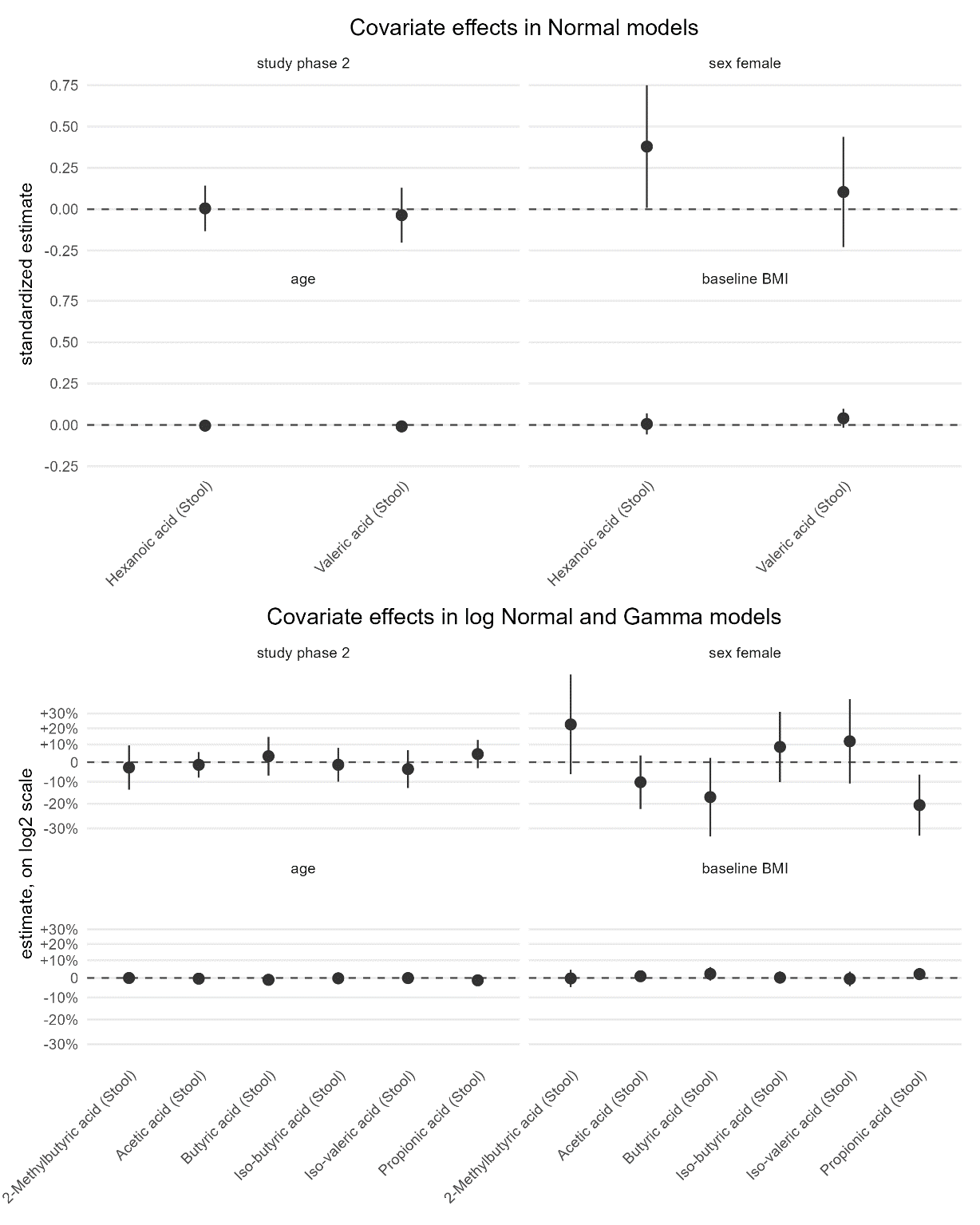


Figure S10: Estimated control variable effects on fecal SCFA levels, including uncorrected 95% confidence intervals; top plot: additive effects based on Normal regression. Estimates were standardized by the respective marker’s standard deviation. The depicted change of 0.1 standard deviations refers to a change of ~9.4 µg/g (valeric acid) and ~12.4 µg/g (hexanoic acid); bottom plot: multiplicative, exponentiated intervention effects on markers based on log Normal or Gamma regression, depicted on a log2-transformed y-axis.


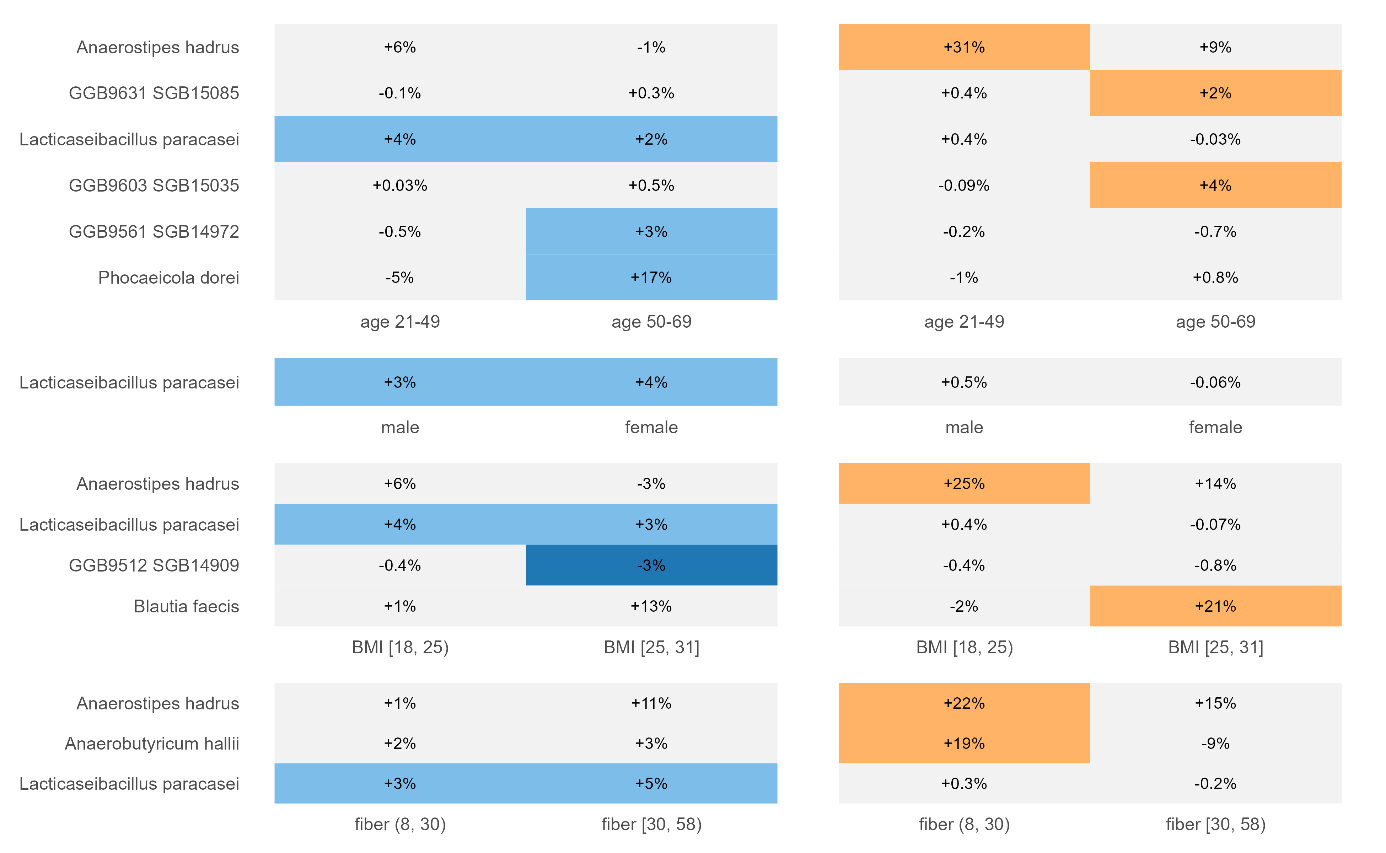


Figure S11: Estimated intervention effects on species’ relative abundance following stratification by age, sex, BMI, and daily fiber intake (q value < 0.1).


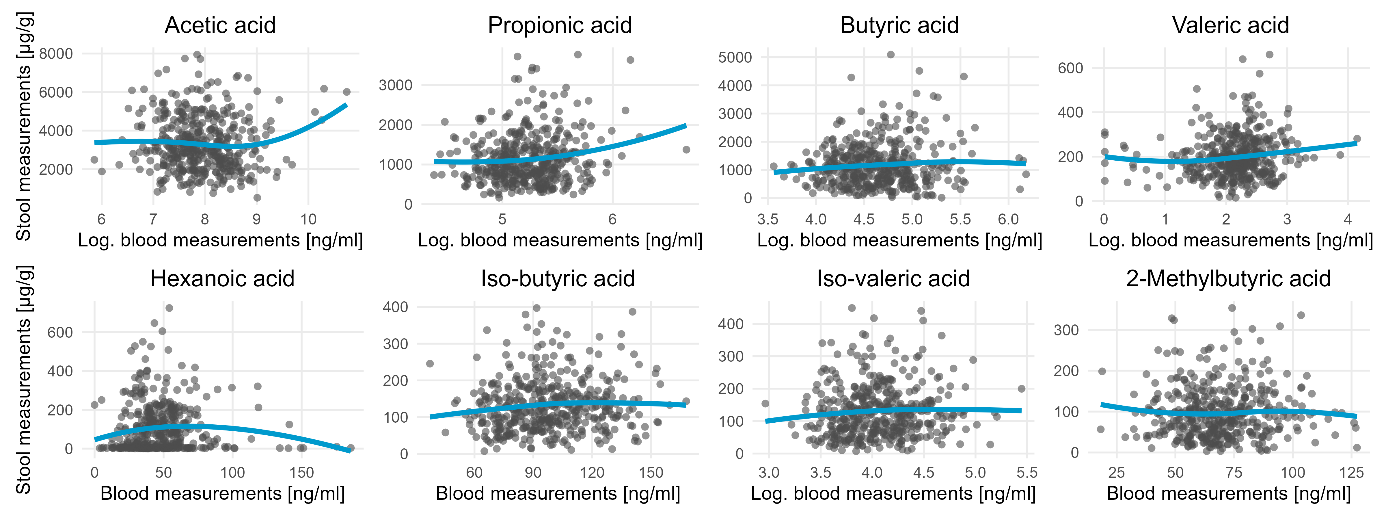


Figure S12: Correlation of SCFA levels in serum and stool


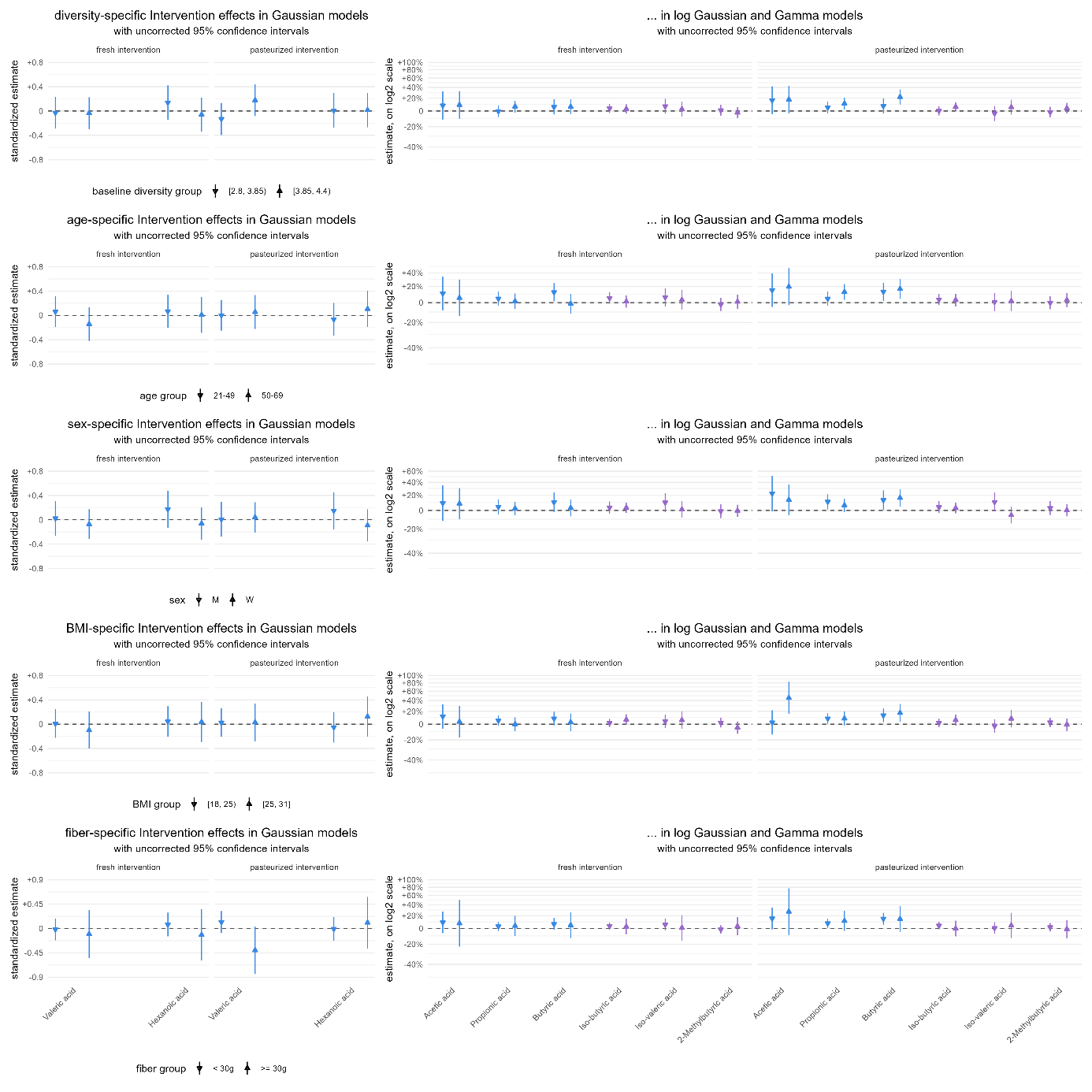


Figure S13: Estimated intervention effects on serum SCFA level stratified by (from top to bottom) baseline Shannon diversity, age, sex, BMI, and daily fiber intake. Effects are shown accompanied by 95% confidence intervals which are uncorrected for multiple testing and should thus be interpreted with some caution.


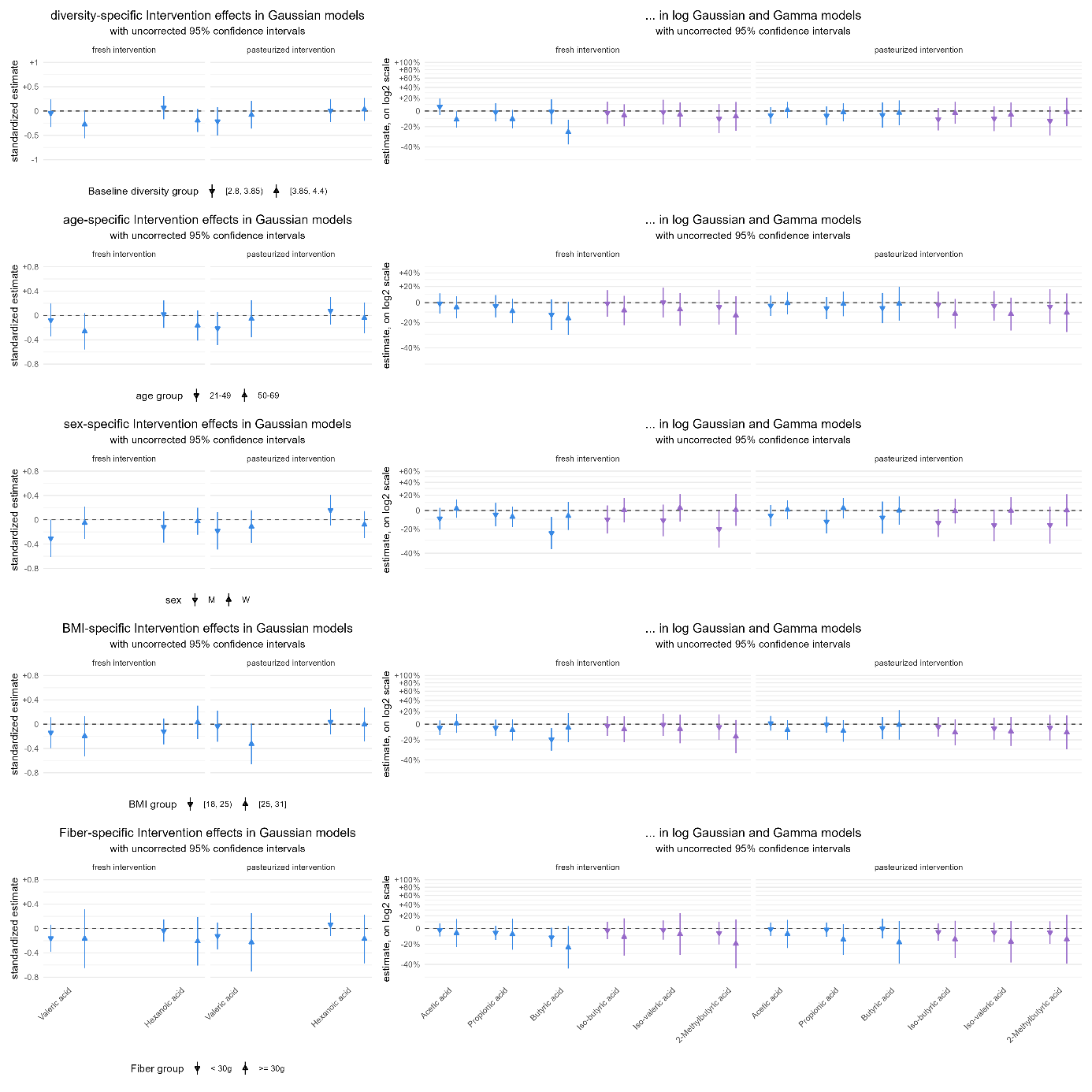


Figure S14: Estimated intervention effects on fecal SCFA level stratified by (from top to bottom) baseline Shannon diversity, age, sex, BMI, and daily fiber intake. Effects are shown accompanied by 95% confidence intervals which are uncorrected for multiple testing and should thus be interpreted with some caution.


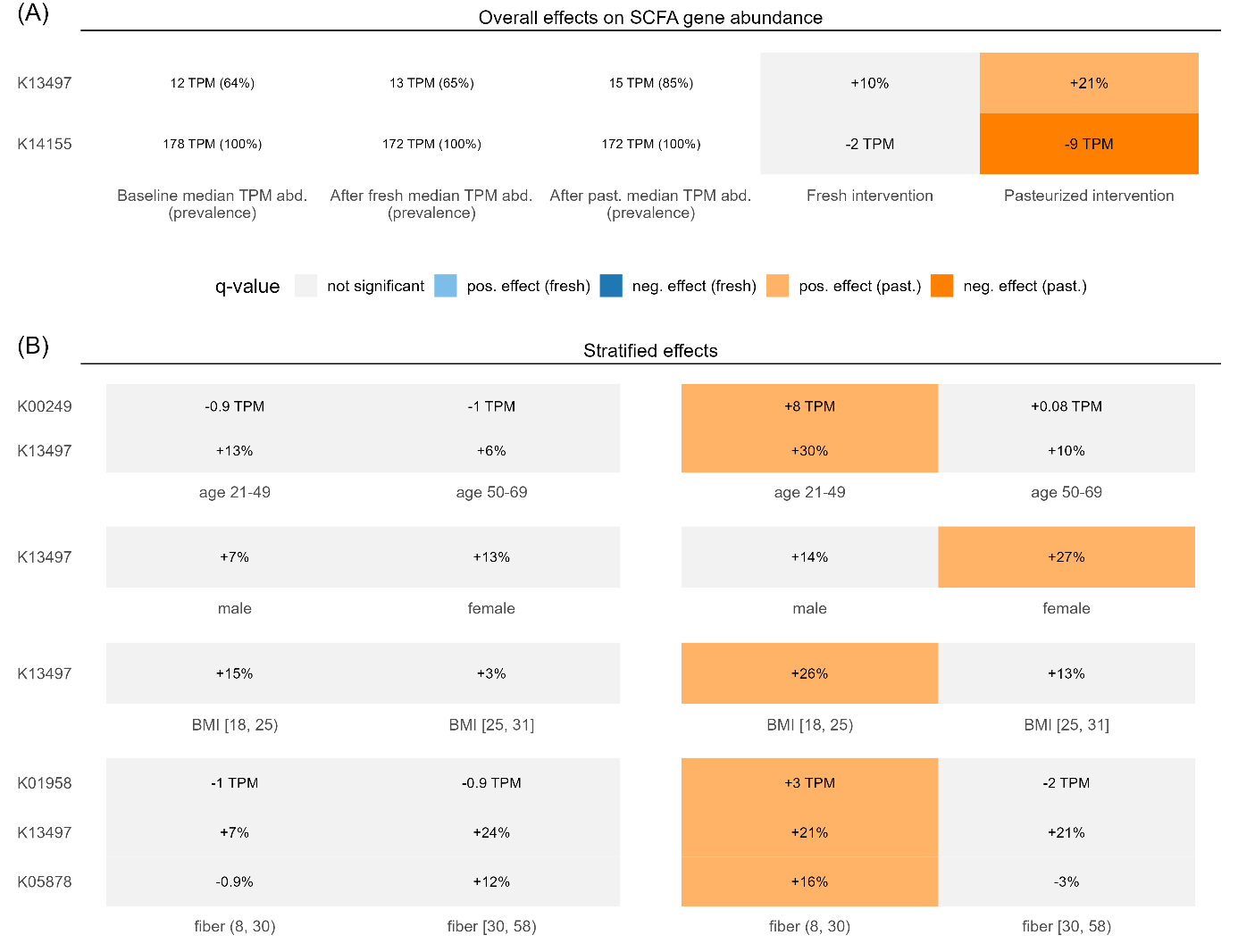


Figure S15: Estimated intervention effects on the relative abundance of SCFA-related KOs following stratification by age, sex, BMI, and daily fiber intake (q value < 0.1).


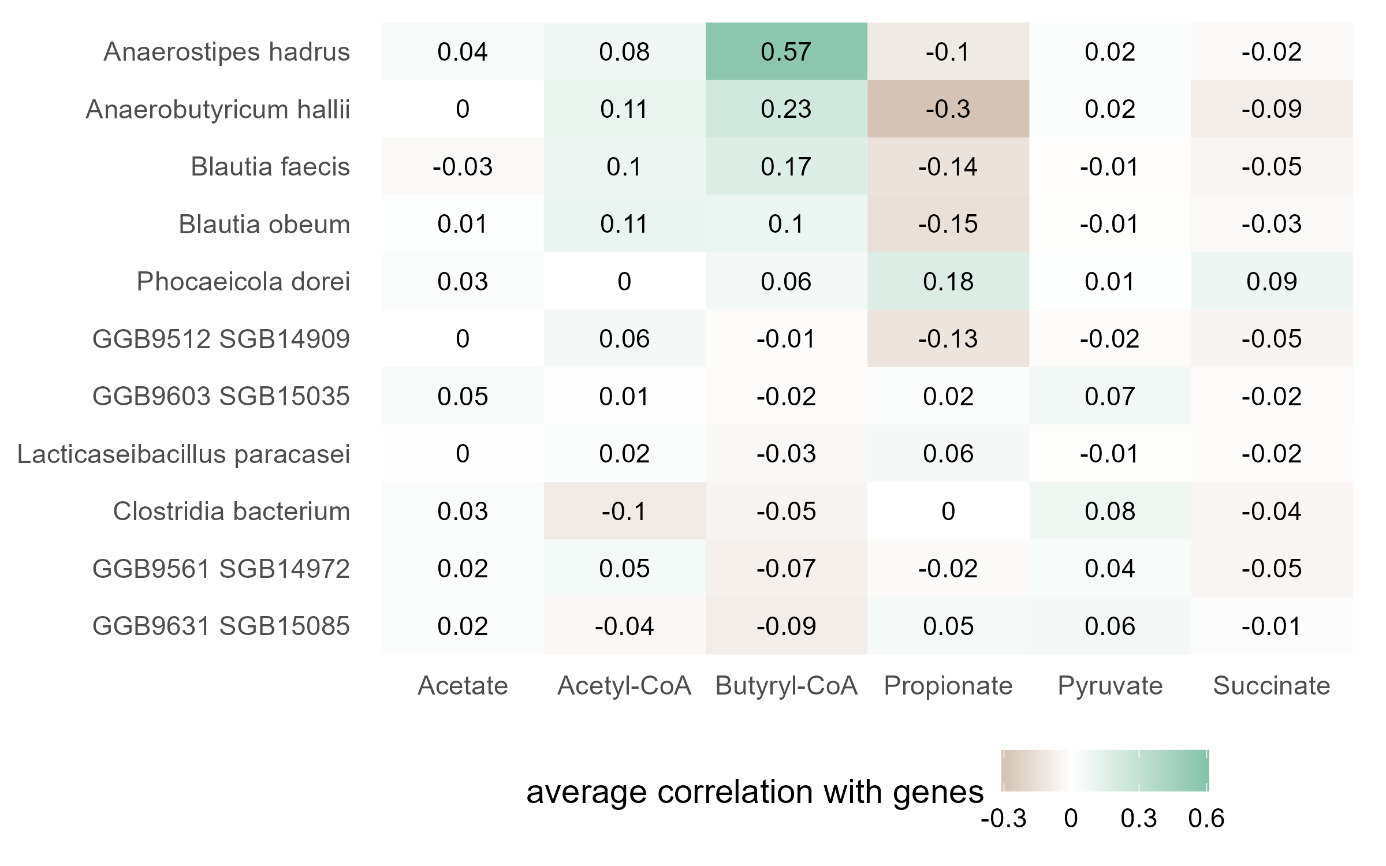


Figure S16: Correlation of species that changed significantly during an intervention in this study and KEGG orthologues (KOs) relevant for SCFA metabolism. KOs are grouped by their association with specific products.


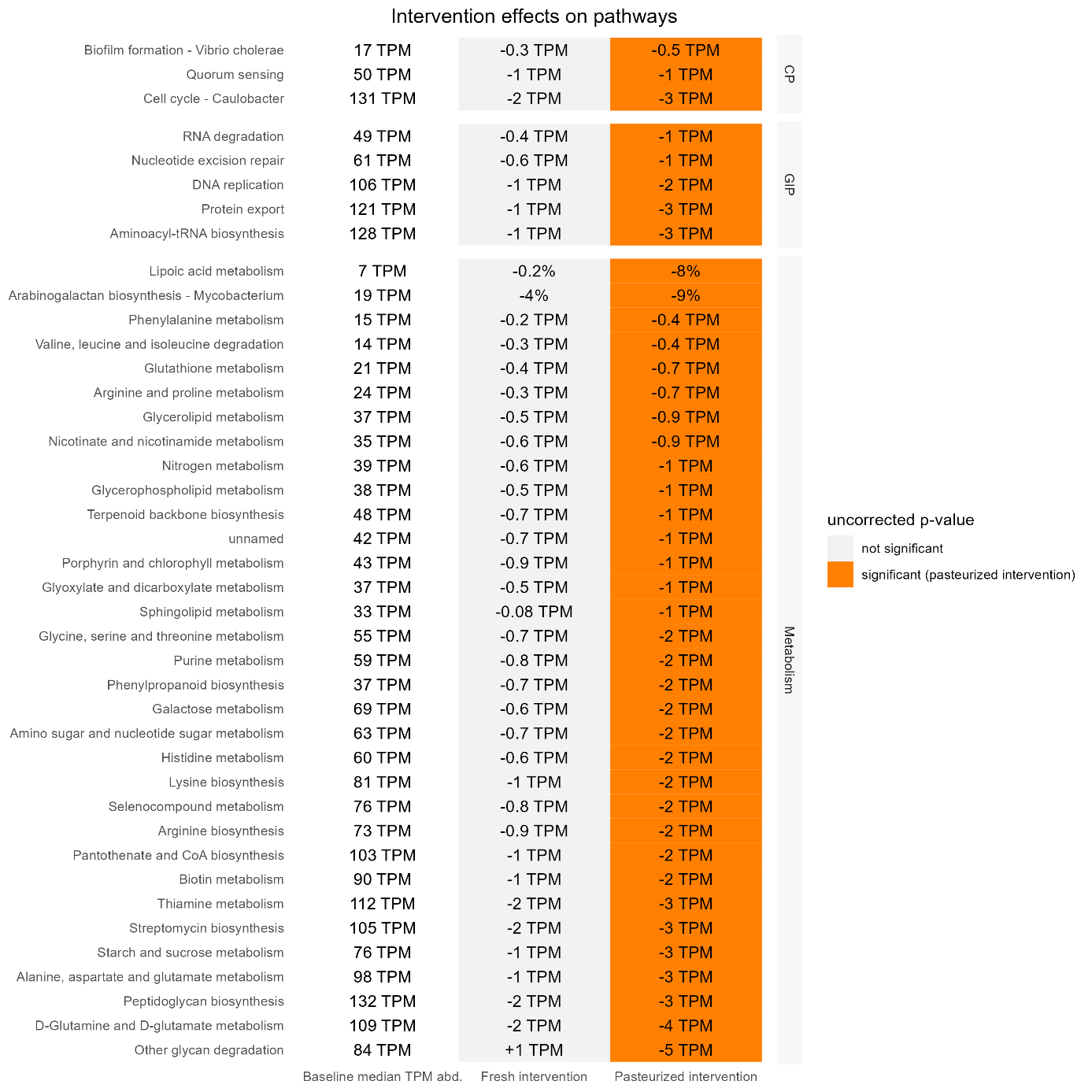


Figure S17: Estimated intervention effects on KEGG pathways’ relative abundance. Effects highlighted in orange had p-values < 0.05 (not corrected for multiple testing) which display trends but should be interpreted with some caution. Pathway group abbreviations: “CP” = Cellular Processes, “GIP” = Genetic Information Processing.
